# The Endophytic Root Microbiome Is Different in Healthy and Ralstonia solanacearum-Infected Plants and Is Regulated by a Consortium Containing Beneficial Endophytic Bacteria

**DOI:** 10.1128/spectrum.02031-22

**Published:** 2022-12-14

**Authors:** Yiting Li, Gaofu Qi, Ziqiong Xie, Baolong Li, Rui Wang, Jun Tan, Heli Shi, Bikun Xiang, Xiuyun Zhao

**Affiliations:** a College of Life Science and Technology, Huazhong Agricultural University, Wuhan, China; b Enshi Tobacco Company of Hubei Province, Enshi, China; Yeungnam University

**Keywords:** *Ralstonia solanacearum*, endophytic root microbiome, microbial network, synthetic microbial consortia, bacillaene, induced systemic resistance

## Abstract

Plant bacterial wilt disease caused by Ralstonia solanacearum leads to huge economic losses worldwide. Endophytes play vital roles in promoting plant growth and health. It is hypothesized that the endophytic root microbiome and network structure are different in healthy and diseased plants. Here, the endophytic root microbiomes and network structures of healthy and diseased tobacco plants were investigated. Composition and network structures of endophytic root microbiomes were distinct between healthy and diseased plants. Healthy plants were enriched with more beneficial bacteria and bacteria with antagonistic activity against R. solanacearum. R. solanacearum was most abundant in diseased plants. Microbial networks in diseased plants had fewer modules and edges, lower connectivity, and fewer keystone microorganisms than those in healthy plants. Almost half of the nodes were unique in the two networks. *Ralstonia* was identified as a key microorganism of the diseased-plant network. In healthy plants, abundant bacteria and biomarkers (Pseudomonas and *Streptomyces*) and keystone microorganisms (*Bacillus*, *Lysobacter*, and *Paenibacillus*) were plant-beneficial bacteria and showed antibacterial and plant growth-promoting activities. The endophytic strain Bacillus velezensis E9 produced bacillaene to inhibit R. solanacearum. Consortia containing keystone microorganisms and beneficial endophytic bacteria significantly regulated the endophytic microbiome and attenuated bacterial wilt by inducing systemic resistance and producing antibiotic. Overall, the endophytic root microbiome and network structure in diseased plants were different from those in healthy plants. The endophytic root microbiome of diseased plants had low abundances of beneficial bacteria and an unstable network and lacked beneficial keystone microorganisms, which favored infection. Synthetic microbial consortia were effective measures for preventing R. solanacearum infection.

**IMPORTANCE** Bacterial wilt disease causes heavy yield losses in many crops. Endophytic microbiomes play important roles in control of plant diseases. However, the role of the endophytic root microbiome in controlling bacterial wilt disease is poorly understood. Here, differences in endophytic root microbiomes and network structures between healthy and diseased tobacco plants are reported. A synthetic microbial consortium containing beneficial endophytic bacteria was used to regulate the endophytic microbiome and attenuate bacterial wilt disease. The results could be generally used to guide control of bacterial wilt disease.

## INTRODUCTION

Plant bacterial wilt disease is a soilborne disease caused by the pathogen Ralstonia solanacearum. R. solanacearum spreads quickly through soil, water, and agricultural operations and causes heavy yield losses in many crops (e.g., tomato, pepper, potato, tobacco, etc.). The main symptoms are sudden wilt and death of the plant; the infected stem turns brown and oozes a slimy bacterial exudate (1). High temperature and high humidity, continuous monocropping, soil acidification, and excessive application of nitrogen fertilizer generally aggravate disease incidence (2). Nowadays, bacterial wilt disease tends to increase year by year, and the scope of this disease has become wider and wider (3). Bacterial wilt disease occurs mainly in southern China, represented by Fujian, Guangxi, Guangdong, Sichuan, Chongqing, Hunan, Hubei, Guizhou and Yunnan provinces, causing extensive destruction to crops (3).

An in-depth understanding of the interactions between endophytes and plant pathogens will help to improve plant resistance through regulation of the endophytic microbiome. Microbial network analysis can discover keystone microorganisms, which maintain the stability and balance of the microbial community and play important roles in inhibiting the growth of plant pathogens (4). In order to reduce the use of chemical pesticides, people have tried to control plant diseases by regulating rhizospheric microbiome. Studies have shown that healthy soil has a specific microbial population that improves plant resistance against infection. Healthy soil has a high abundance of genes related to the synthesis of antibacterial substances (5). Endophytes contribute to host resistance against pathogens via the induction of host resistance genes, competition, and production of bioactive compounds (6). Therefore, endophytic microbiomes play important roles in the control of plant diseases.

Individual antagonistic microorganisms are rarely able to eradicate plant soilborne diseases (7). Therefore, determining how to effectively antagonize pathogens through a consortium of microbial agents is very important (8). Microbial consortia can perform complex functions that individual microorganisms cannot. Synthetic or complex microbial consortia are more effective in controlling diseases than single strains (9). Few reports describe the application of synthetic microbial consortia to control bacterial wilt disease (10). Keystone microorganisms of the endophytic microbiome may be good candidates for constructing microbial consortia.

The role of endophytic root microbiome and microbial interactions in controlling bacterial wilt disease is poorly understood. This study compared the endophytic root microbiomes and network structures of diseased and healthy tobacco plants. It was hypothesized that the endophytic microbiomes would be different between the healthy and diseased plants and that beneficial endophytes, including endophytic bacteria with antagonistic activity against pathogens, would be enriched in healthy plant roots at the onset of the infection. Synthetic microbial consortia containing plant-beneficial endophytes might control bacterial wilt disease. Multiple mechanisms, including induced systemic resistance (ISR), production of antibacterial compounds, biofilm formation, and promotion of plant growth, might improve plant resistance against bacterial wilt disease.

## RESULTS

### Endophytic root microbiomes are different in healthy and diseased plants.

**FIG 1:**
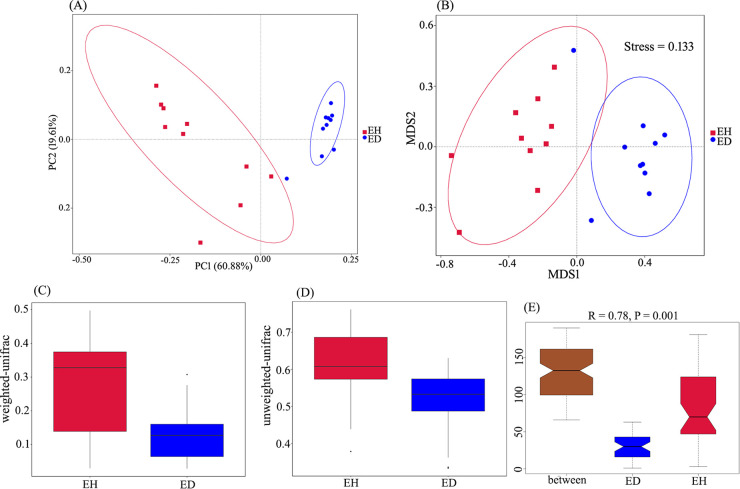
Comparison of endophytic root microbiome structure and beta diversity index between diseased plants (ED) and healthy plants (EH). (A) PCoA analysis. (B) NMDS analysis. (C) Weighted UniFrac beta analysis. (D) Unweighted UniFrac beta analysis. (E) ANOSIM of community structure differences between the two groups.

The endophytic root microbiomes of diseased and healthy Nicotiana tabacum plants were compared by 16S rRNA amplicon sequencing. Principal-coordinate analysis (PCoA) and nonmetric multidimensional scaling (NMDS) analysis showed that the structure of the endophytic root microbiome in the diseased plants was different from that in healthy plants (Fig. 1A and B). The species richness of the diseased-plant microbiome was lower than that of the healthy-plant microbiome. Species diversity in the diseased-plant microbiome was significantly (*P < *0.01) different from that of the healthy-plant microbiome, as revealed by comparing the beta diversity indices of two groups (Fig. 1C and D). Analysis of similarity (ANOSIM) showed a significant difference (*R* = 0.78, *P = *0.001) between the two microbiomes (Fig. 1E). Overall, endophytic root microbiomes were different between healthy and diseased plants.

### The endophytic root microbiome composition of diseased plants is different from that of the healthy-plant microbiome.

**FIG 2:**
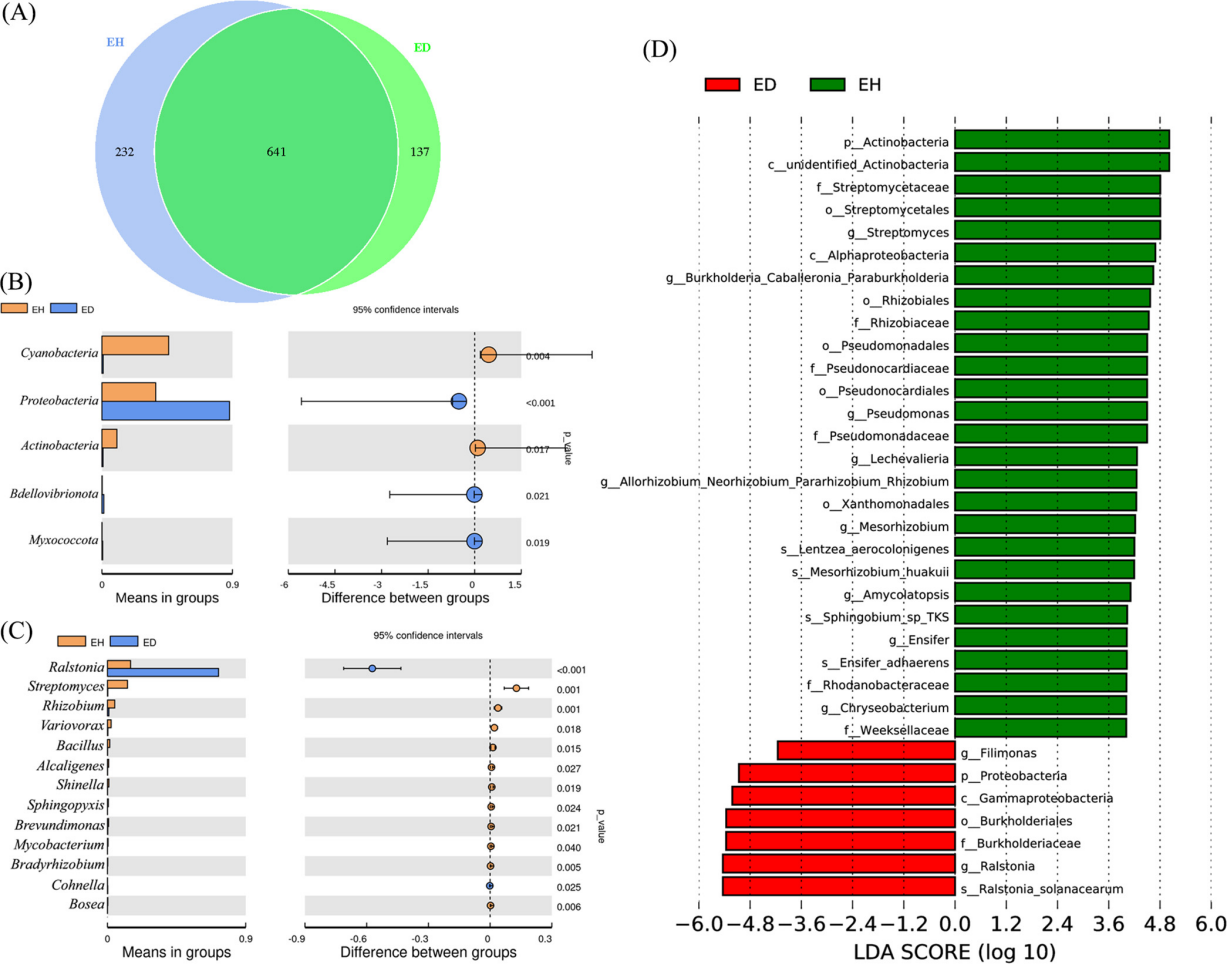
Analysis of species differences between the endophytic root microbiome of healthy (EH) and diseased (ED) plants. (A) Venn diagram showing the shared and unshared OTUs among endophytic root microbiomes of healthy and diseased plants. (B and C) *t*-test analysis of phylum (B) and genus (C) differences between the two groups. (D) LEfSe analysis.

Sequences with ≥97% similarity were assigned to the same operational taxonomic units (OTUs). OTUs of two microbiomes were analyzed by jvenn. A total of 641 OTUs (77.6%) were shared by the endophytic root microbiomes of diseased and healthy plants (Fig. 2A). The healthy-plant microbiome had more unique OTUs (232 OTUs) than the diseased-plant microbiome (137 OTUs). Relative abundances of *Actinobacteria* and *Cyanobacteria* in the diseased plants were significantly (*P < *0.05) lower than those in healthy plants (Fig. 2B).

The difference in genus abundance between the two groups was compared by *t*-test analysis. The relative abundances of 13 genera were significantly (*P < *0.05) different between the two groups (Fig. 2C). Relative abundance of *Ralstonia* in diseased plants (71.02%) was significantly (*P < *0.01) higher than in healthy plants (5.67%), which included the pathogen R. solanacearum, which causes bacterial wilt disease. The relative abundances of 11 genera (i.e., *Alcaligenes*, *Bacillus*, *Bosea*, *Bradyrhizobium*, *Brevundimonas*, Mycobacterium, *Rhizobium*, *Shinella*, *Sphingopyxis*, *Streptomyces*, and *Variovorax*) in the diseased plant were significantly (*P < *0.05) lower than in healthy plants.

The core microbes were identified by linear discriminant analysis effect size (LEfSe) analysis. The core microbial populations in healthy plant consisted of plant-beneficial bacteria such as *Ensifer*, *Mesorhizobium*, Pseudomonas, and *Streptomyces*, which possibly promoted plant growth and inhibited infection (Fig. 2D). The core microbial species of the diseased-plant microbiome was identified as R. solanacearum, which led to withering of most plants (70.72%).

### The microbial network structure differs between healthy and diseased plants.

**FIG 3:**
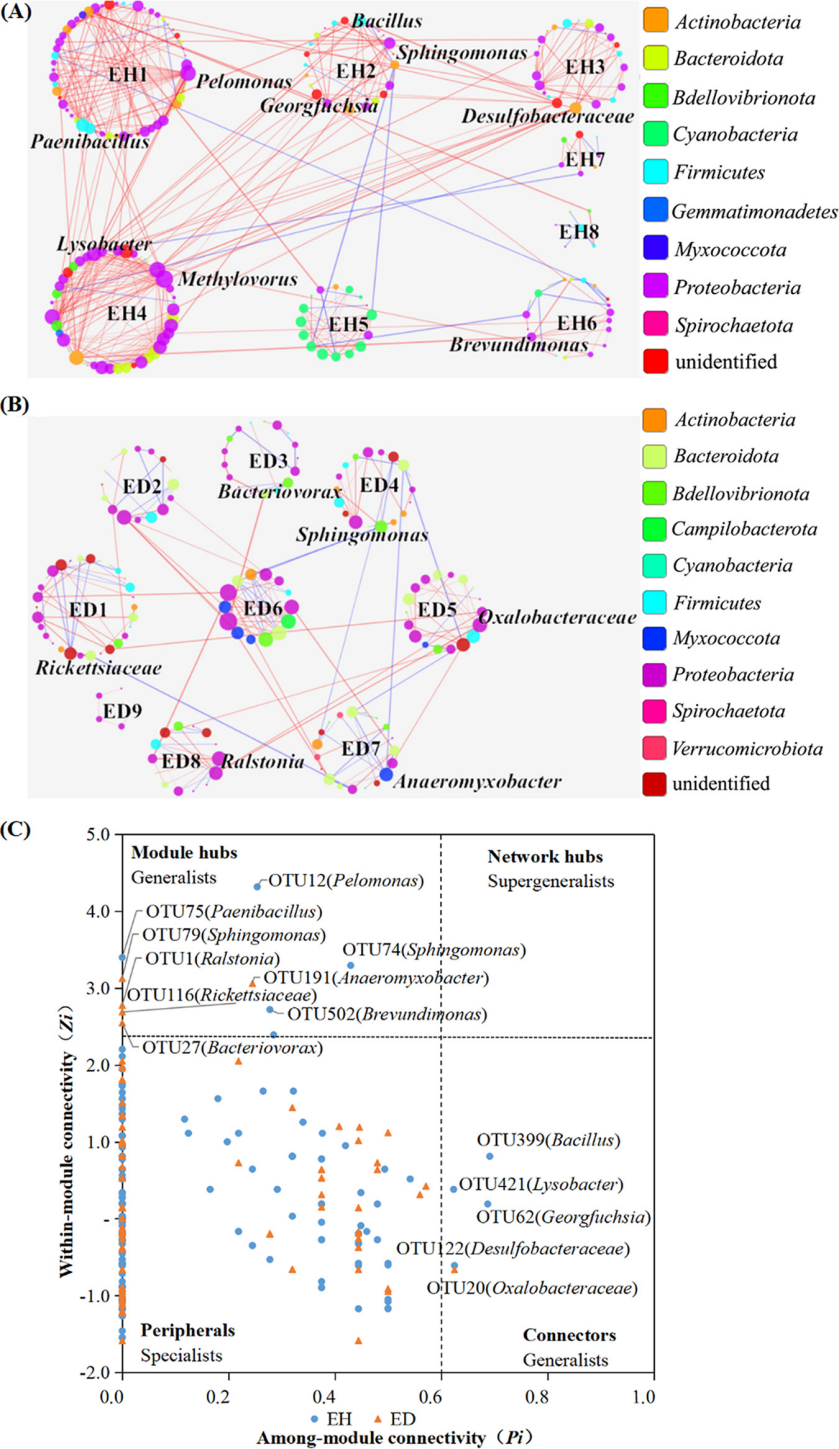
Overview of microbial networks and topological roles. (A and B) Graphs of endophytic networks of healthy plants (A) and diseased plants (B). (C) Topological roles of nodes. Threshold values of *Z_i_* and *P_i_* for categorizing nodes were 2.5 and 0.62, respectively. Keystone microorganisms are labeled with OTU number and phylogenetic relationship. ED, diseased-plant network; EH, healthy-plant network.

Microbial networks were constructed by the Molecular Ecological Network Analysis Pipeline. Microbial networks of healthy and diseased plants contained 8 and 9 modules (≥5 nodes) (Fig. 3A and B). The healthy-plant network (228 nodes and 519 edges) contained more connections and nodes than the diseased-plant network (198 nodes and 257 edges), indicating that microbial interactions in diseased plants decreased compared to those in healthy plants (Table 1). The clustering coefficients, connectivity, and modularity of the two networks were all different from each other, which indicated that the topological properties of the endophytic network were different between healthy and diseased plants. Average connectivity (avgK) was used to assess network complexity. The networks of healthy plants (avgK, 4.553) were more complex than those of diseased plants (avgK, 2.596). The average geodesic distance of healthy-plant networks (5.264) was lower than that of diseased-plant networks (6.81); the average clustering coefficient (0.22) of healthy-plant networks was higher than that of diseased-plant networks (0.116), which indicated that nodes of healthy-plant networks were closer than nodes of diseased-plant networks. Connectivity and clustering coefficients of diseased-plant networks were lower than those of healthy-plant networks, indicating that the stability of diseased-plant networks was lower than that of healthy-plant networks. These results suggested that microbial network structure in diseased plants was different from that in healthy plants.

**TABLE 1:** Microbial network structure of endophytic root microbiome

Condition	Empirical networks^*a*^						Random networks (avg ± SD)		
	*S_t_^b^*	Network size (no. of nodes)	Avg connectivity	Avg geodesic distance	Avg clustering coefficient	Modularity (no. of modules)	Geodesic distance	Clustering coefficient	Modularity
EH	0.84	228	4.553	5.264	0.22	0.646 (19)	3.517 ± 0.051	0.042 ± 0.008	0.436 ± 0.007
ED	0.81	198	2.596	6.81	0.116	0.802 (27)	4.964 ± 0.134	0.012 ± 0.007	0.665 ± 0.011

a *S_t_^b^*, similarity threshold.

The nodes of two networks were compared by jvenn. Most nodes belonged to nine phyla (see Fig. S1A in the supplemental material). Proportions of *Actinobacteria*, *Cyanobacteria*, *Firmicutes*, *Spirochaetota*, and *Proteobacteria* in the diseased-plant network were lower than in the healthy-plant network. One hundred fifteen nodes were shared by two networks (Fig. S1B). The diseased-plant network had fewer specific nodes than the healthy-plant network. These results indicated that the composition of the microbial network was different in the healthy and diseased plants.

The topological roles of nodes were different between healthy and diseased plants. These roles were defined by the within-module connectivity (*Z_i_*) and among-module connectivity (*P_i_*) (11) (Fig. 3C). The majority of nodes (96.5%) were peripherals (referred to as specialists). Connectors were highly connected with other modules. Module hubs were highly connected with many nodes in their own modules. Of the nodes, 3.5% were module hubs (10 nodes) and connectors (5 nodes) (both referred to as generalists) in the two networks. No network hub (supergeneralist) was found in the two networks. Module hubs, connectors, and network hubs were the keystone microorganisms. More keystone microorganisms (5 module hubs and 4 connectors) were found in the healthy-plant network than in the diseased-plant network (5 module hubs and 1 connector). Nine keystone microorganisms of the healthy-plant network belonged to *Firmicutes* and *Proteobacteria*. Four module hubs (OTU12, OTU17, OTU74, and OTU502) belonging to *Proteobacteria* were closely related to *Pelomonas*, *Methylovorus*, *Sphingomonas*, and *Brevundimonas*, respectively. One module hub (OTU75) belonging to *Firmicutes* was closely related to Paenibacillus turicensis. Three connectors (OTU62, OTU122, and OTU421) belonging to *Proteobacteria* were closely related to *Georgfuchsia*, *Desulfobacteraceae*, and *Lysobacter*, respectively. One connector (OTU399) belonging to *Firmicutes* was closely related to *Bacillus*. These results indicated that plant-beneficial endophytic bacteria (i.e., *Bacillus*, *Brevundimonas*, *Lysobacter*, and *Paenibacillus*) played key roles in stabilizing the healthy-plant network.

Keystone microorganisms of the diseased-plant network were analyzed. Six keystone microorganisms of the diseased-plant network belonged to *Bdellovibrionota*, *Myxococcota*, and *Proteobacteria*. Three module hubs (OTU1, OTU79, and OTU116) belonging to *Proteobacteria* were closely related to R. solanacearum, *Sphingomonas*, and *Rickettsiaceae*, respectively. One module hub (OTU27) belonging to *Bdellovibrionota* was closely related to *Bacteriovorax*. One module hub (OTU191) belonging to *Myxococcota* was closely related to *Anaeromyxobacter*. One connector (OTU20) belonging to *Proteobacteria* was closely related to *Oxalobacteraceae*. These results indicated that the keystone microorganisms of the two networks were different. R. solanacearum was identified as one of keystone microorganisms in the diseased-plant network, suggesting that this pathogen occupied an important ecological niche and affected network function. It is worth noting that some nodes (e.g., OTU12, OTU17, OTU62, OTU122, and OTU399) were identified as generalists in the healthy-plant network but as specialists in diseased-plant network. In turn, some nodes (OTU1, OTU20, OTU27, OTU79, and OTU116) were identified as generalists in the diseased-plant network but as specialists in the healthy-plant network. This suggested that the same endophytic bacterial species play different roles in the two networks.

Correlation between keystone microorganisms and disease severity index was analyzed using SPSS software. Many keystone microorganisms were significantly (*P < *0.05) correlated with disease severity index (Table 2). In the diseased-plant network, R. solanacearum (OTU1), *Oxalobacteraceae* (OTU20), and *Sphingomonas* (OTU79) were significantly positively correlated with disease severity index, indicating that they might aggravate tobacco bacterial wilt disease.

**TABLE 2:** Correlations between key microorganisms and disease severity index

Key microorganism	Correlation (*P*)
OTU17 (*Methylovorus*)	0.441 (0.051)
OTU62 (*Georgfuchsia*)	−0.122 (0.609)
OTU75 (*Paenibacillus*)	0.438 (0.054)
OTU122 (*Desulfobacteraceae*)	0.425 (0.062)
OTU421 (*Lysobacter*)	0.366 (0.113)
OTU502 (*Brevundimonas*)	−0.336 (0.147)
OTU1 (Ralstonia solanacearum)	0.740 (0.0003)^*a*^
OTU20 (*Oxalobacteraceae*)	0.454 (0.044)^*b*^
OTU27 (*Bacteriovorax*)	0.243 (0.301)
OTU79 (*Sphingomonas*)	0.673 (0.001)^*a*^
OTU116 (*Rickettsiaceae*)	−0.293 (0.213)
OTU191 (*Anaeromyxobacter*)	0.441 (0.052)

a Very significant (*P *< 0.01) correlation.

b Significant (*P *< 0.05) correlation.

### Interactions among R. solanacearum and other nodes of the endophytic network.

**FIG 4:**
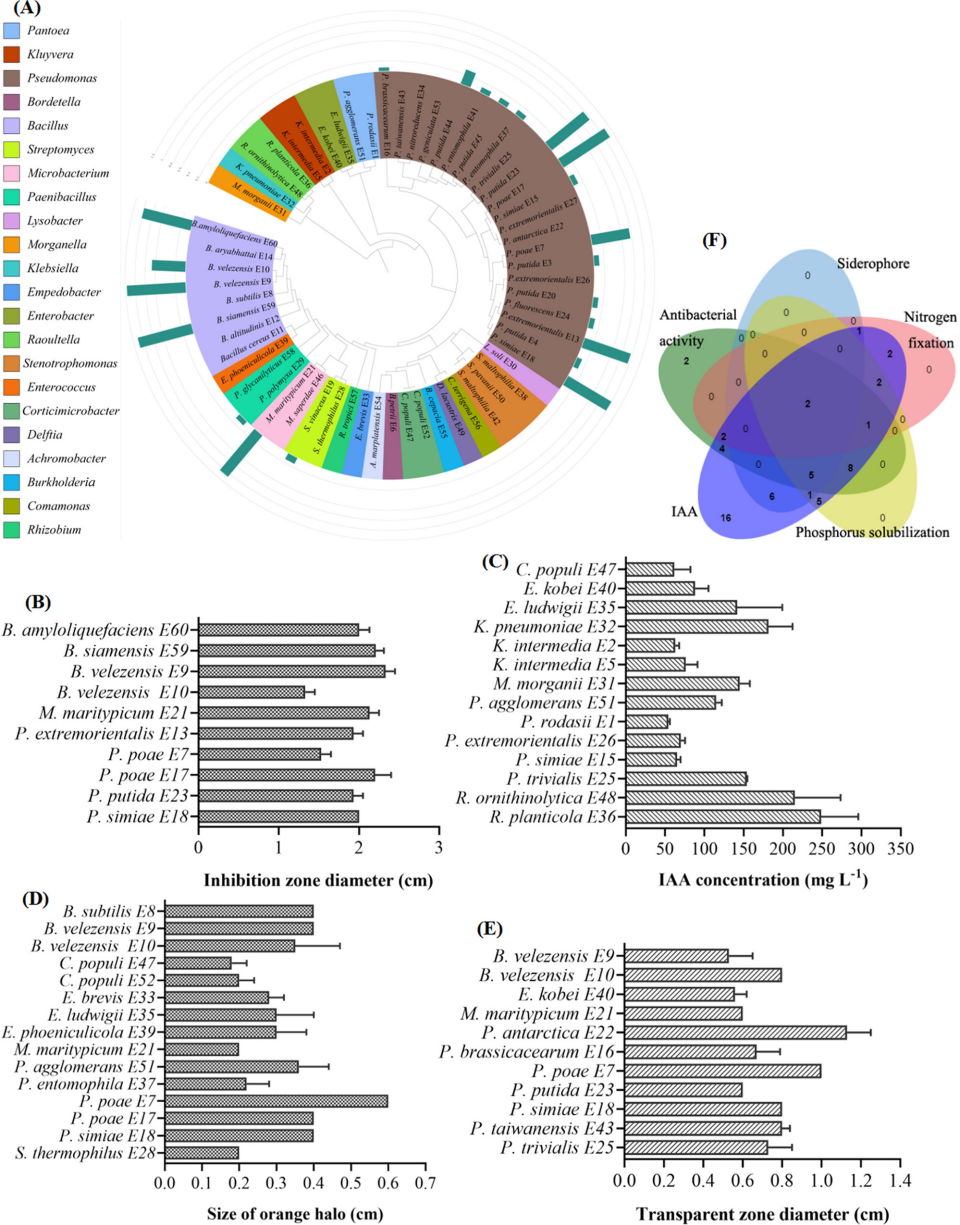
Detecting activities of root endophytic bacteria. (A) Phylogenetic tree of endophytic bacterial isolates. Colors depict the taxonomic classifications. Green bars represent the antibacterial activities of the isolates. Isolates E1 to E30 were isolated from healthy plants. Isolates E31 to E60 were isolated from diseased plants. (B) Antibacterial activities of endophytic bacteria against R. solanacearum. (C) Production of IAA by endophytic bacteria. (D) Production of siderophores by endophytic bacteria. (E) Organic-phosphorus solubilizing activity of endophytic bacteria. (F) Venn diagram indicating activities of beneficial endophytic bacteria. All data are averages with standard deviations from triplicate experiments.

Sixty endophytic bacterial isolates were cultured from tobacco plant roots that were collected for analysis of the endophytic root microbiome. Isolates E1 to E30 were cultured from healthy plants; isolates E31 to E60 were cultured from diseased plants (Fig. 4A). Molecular identification based on 16S rRNA genes showed that the endophytic bacterial isolates belonged to 22 genera. Plant growth-promoting and pathogen-antagonistic endophytic bacteria were isolated from roots. Twenty-four endophytic bacterial isolates inhibited the cell growth of R. solanacearum (Table S1). Antagonistic bacteria belonged to *Bacillus*, *Microbacterium*, *Paenibacillus*, Pseudomonas, and *Streptomyces*. Ten endophytic bacterial isolates showed high antibacterial activity (inhibition zone diameter > 1 cm). Bacillus velezensis E9 had the highest antibacterial activity, followed by Bacillus siamensis E59 (Fig. 4B).

Plant growth-promoting activities of endophytic bacteria were further detected. Indole acetic acid (IAA) is a hormone that promotes plant growth. Sixty endophytic bacterial isolates all used serine to synthesize IAA (3.45 to 248.67 mg/L). Fourteen isolates produced high concentrations of IAA (>50 mg/L) (Fig. 4C); these endophytic bacteria belonged to *Corticimicrobacter*, Enterobacter, Klebsiella, *Kluyvera*, *Morganella*, *Pantoea*, Pseudomonas, and *Raoultella*. Raoultella planticola E36 produced the highest concentration of IAA (248.67 mg/L), followed by *R. ornithinolytica* E48 (215.23 mg/L) and Klebsiella pneumoniae E32 (181.61 mg/L). Fifteen isolates produced siderophores (Fig. 4D); these endophytic bacteria belonged to *Bacillus*, *Corticimicrobacter*, *Empedobacter*, Enterobacter, *Enterococcus*, *Microbacterium*, *Pantoea*, Pseudomonas, and *Streptomyces*. Among them, Pseudomonas poae E7 had the highest activity.

Ten isolates grew well in Ashby medium without a nitrogen source and had potential nitrogen-fixing activity (Fig. S2A). Achromobacter marplatensis E54, Bacillus velezensis E9, Pseudomonas extremorientalis E13, and Pseudomonas simiae E18 genomes contained the nitrogenase gene *nifH* (Fig. S2B, C). Phosphorus-solubilizing activity of endophytic bacteria was detected by the transparent zone method (12). Twenty-four endophytic bacteria belonging to *Bacillus*, Enterobacter, Klebsiella, *Microbacterium*, *Morganella*, Pseudomonas, and *Raoultella* solubilized organic phosphorus (Fig. 4E). Eleven isolates showed higher activity (transparent-zone diameter > 0.5 cm). Pseudomonas antarctica E22 had the strongest phosphate solubilization ability, followed by *P. poae* E7.

Biofilm production plays important roles in the plant root colonization process of endophytic bacteria. Twenty-two endophytic bacterial isolates formed biofilms. Thirteen of these isolates formed denser biofilms (OD_590_ > 2). B. subtilis E8 formed the most biofilm, followed by *B. siamensis* E59 and *B. velezensis* E9 (Fig. S3). Some endophytic bacterial isolates had multiple activities (Fig. 4F). For instance, *B. velezensis* E9 and *P. simiae* E18 showed five activities, including antibacterial activity, nitrogen fixation, phosphorus solubilization, production of IAA, and production of siderophores. *B. velezensis* E10, Microbacterium maritypicum E21, Pseudomonas entomophila E37, *P. poae* E17, and *P. poae* E7 produced siderophores and IAA, solubilized phosphorus, and inhibited R. solanacearum growth.

**FIG 5:**
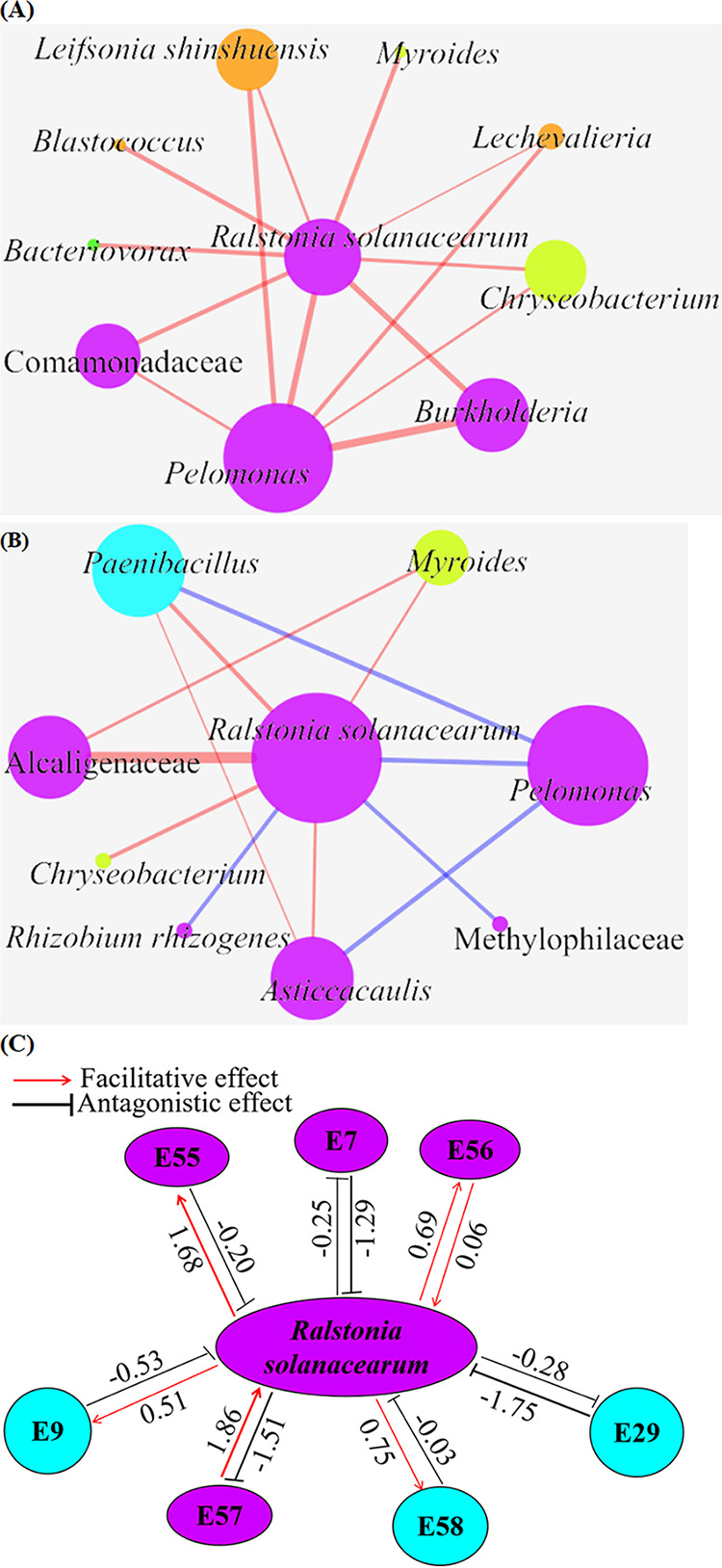
Interaction of R. solanacearum and other nodes in endophytic microbiomes. (A and B) Subnetwork graphs of R. solanacearum and its interacting nodes in endophytic microbiomes of healthy plants (A) and diseased plants (B). Nodes of different colors belong to different bacterial phyla. Blue links represent negative interactions between nodes, and red links represent positive interactions. Modules with five or more nodes are included. Node size is proportionate to the node degree (connectivity of nodes). Edge width is proportionate to the edge betweenness (strength of the correlation). Keystone microorganisms, R. solanacearum, and its interacted nodes are labeled. (C) Analysis of the type of interaction between microorganisms and R. solanacearum by the cocultivation method. The mean intensity of interactions is indicated. The strains used are Bacillus velezensis E9, Bordetella petrii E6, Burkholderia cepacia E55, Comamonas terrigena E56, Empedobacter brevis E33, Paenibacillus glycanilyticus E58, Paenibacillus polymyxa E29, Pseudomonas poae E7, Rhizobium tropici E57, and Stenotrophomonas maltophilia E42.

Interactions among R. solanacearum and other endophytic bacteria were analyzed by constructing subnetworks (Fig. 5A and B). In the subnetwork of healthy plants, R. solanacearum was positively correlated with *Bacteriovorax*, *Blastococcus*, *Burkholderia*, *Chryseobacterium*, *Comamonadaceae*, *Lechevalieria*, Leifsonia shinshuensis, *Myroides*, and *Pelomonas* (Fig. 5A). In the subnetwork of diseased plants, R. solanacearum was positively correlated with *Alcaligenaceae*, *Asticcacaulis*, *Chryseobacterium*, *Myroides*, and *Paenibacillus*, while it was negatively correlated with *Methylophilaceae*, *Pelomonas*, and Rhizobium rhizogenes (Fig. 5B), indicating that interactions among R. solanacearum and other microbial members varied in the two networks. Endophytic bacteria might assist or inhibit pathogen infection.

Interactions between R. solanacearum and other endophytic bacteria were verified by cocultivation (Table S2). Thirty-one endophytic bacteria showed antagonistic effects on R. solanacearum. Bacillus amyloliquefaciens E60, *B. velezensis* E9, Corticimicrobacter populi E47, Paenibacillus polymyxa E29, *P. poae* E7, *P. antarctica* E22, and Stenotrophomonas maltophilia E38 showed strong antagonistic effects on R. solanacearum. Nineteen endophytic bacterial isolates showed facilitative effects on R. solanacearum. The cocultivation method correctly reflected interactions between microorganisms and supported the findings of microbiome analysis (Fig. 5C). As the keystone microorganisms of the healthy-plant microbiome, *B. velezensis* E9 and *P. polymyxa* E29 suppressed R. solanacearum.

### An antibacterial substance produced by Bacillus velezensis E9 was identified as bacillaene.

Biosynthesis gene encoding antibacterial substance was analyzed by a gene knockout method. It has been shown that both nonribosomal synthesis of cyclic lipopeptides and synthesis of polyketides are dependent on the presence of a functional *sfp* gene product, 4′-phosphopantetheinyl transferase (13). First, we tested whether antibacterial activity of a Δ*sfp* E9 mutant decreased. Second, E9 mutants with disruption of genes involved in synthesis of bacilysin (Δ*bacE*), surfactin (Δ*srfA*), bacillibactin (Δ*ymfD*), and bacillaene (Δ*acpK*, Δ*baeD*, and Δ*pksG*) were constructed (Fig. S4). Bacilysin is a nonribosomally synthesized dipeptide antibiotic that is active against a wide range of bacteria and fungi (14). Surfactin is a cyclic lipopeptide synthesized by *Bacillus* (15). Bacillibactin is hexadentate catecholate siderophore produced by bacteria upon iron limitation to scavenge ferric ion (16). Bacillaene is a polyene antibiotic produced by *Bacillus* that inhibits prokaryotic growth by disrupting protein synthesis (17).

**FIG 6:**
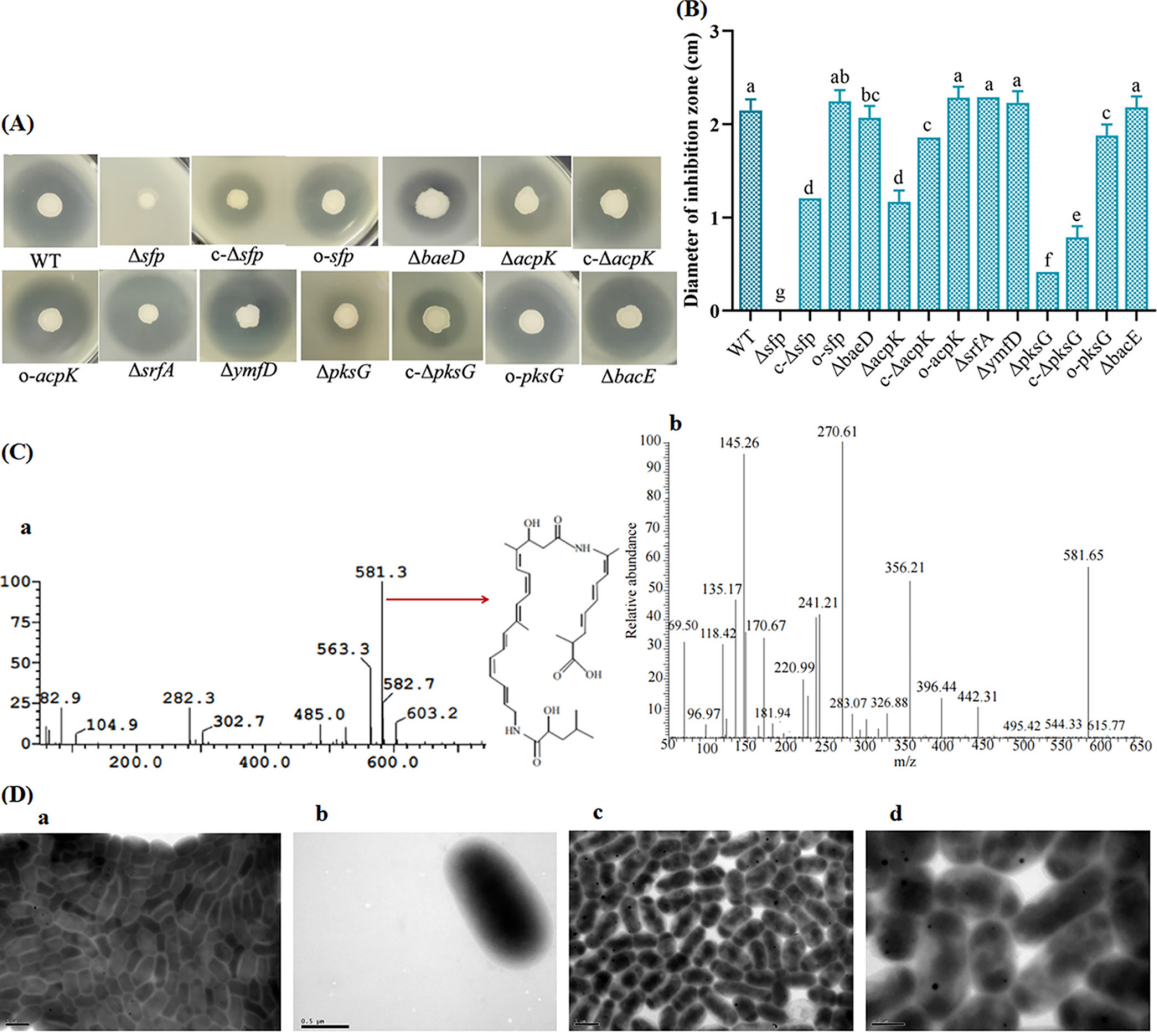
(A and B) Antibacterial activities of *B. velezensis* E9 wild-type (WT) strain and mutants against R. solanacearum. (B) Data are averages with standard deviations from triplicate experiments. Different letters indicate significant (*P < *0.05) differences between strains. “c-” indicates a complemented strain; “o-” indicates an overexpressing strain. (C) Positive-ion electrospray ionization mass spectrometry (ESI-m) (*m/z*) of the antibacterial substance (a) and the secondary mass spectrum of bacillaene (b). The molecular formula of bacillaene is indicated with an arrow. (D) Effect of bacillaene on cell morphology of R. solanacearum. (a and b) Cell morphology of the control group at 24 h (bars, 500 nm [a] and 1 μm [b]). (c and d) Morphology of R. solanacearum cells after treatment with bacillaene for 24 h (bars, 500 nm [c] and 1 μm [d]).

The activities of mutants against R. solanacearum were detected by inhibition zone experiments. Antibacterial activities of Δ*sfp*, Δ*acpK*, Δ*baeD*, and Δ*pksG* mutants were significantly (*P < *0.05) lower than that of the E9 wild-type strain (WT) (Fig. 6A and B). The diameters of the inhibition zones of the Δ*sfp*, Δ*acpK*, Δ*baeD*, and Δ*pksG* mutants decreased by 100%, 46.9%, 12.2%, and 81.2%, respectively, compared to those of the wild-type strain. The synthesis of antibacterial antibiotics in *B. velezensis* E9 was dependent on the presence of the *sfp* product, 4′-phosphopantetheinyl transferase, as evidenced by the fact that a knockout mutation of *sfp* resulted in complete loss of antibacterial activity. Antibacterial substances were related to ribosomal and nonribosomal peptides and polyketones. When the ribosomal- and nonribosomal-peptide-related genes *srfA* and *ymfD* were knocked out, the antibacterial activities of the Δ*srfA* and Δ*ymfD* mutants did not change, indicating that the antibacterial substances were not ribosomal and nonribosomal peptides.

*acpK* encodes AcpK, which is involved in the synthesis of bacillaene (18). *baeD* encodes bacillaene synthase *trans*-acting acyltransferase. *pksG* encodes acetyl-*S*-AcpK beta-ketothioester bacillaene intermediate transferase. It was found that *acpK*, *baeD*, and *pksG* were all involved in synthesis of antibacterial substance in *B. velezensis* E9. The antibacterial substance was mainly the polyketide bacillaene. Complementation of the Δ*sfp*, Δ*acpK*, and Δ*pksG* mutants partially restored antibacterial activity. Complementation of the mutant strains (c-Δ*sfp*, c-Δ*acpK*, and c-Δ*pksG*) restored 56.3%, 84.5%, and 34.3% of antibacterial activity, respectively. Overexpression of two genes (o-*sfp* and o-*acpK*) resulted in antibacterial activity as high as that of the wild-type strain (*P > *0.05). A strain overexpressing *pksG* (o-*pksG*) exhibited significantly (*P < *0.05) higher antibacterial activity than the Δ*pksG* mutant and the c-Δ*pksG* strain, while antibacterial activity of the o-*pksG* strain was lower than that of wild-type strain. These results further verified that the antibacterial substance produced by E9 was bacillaene.

The antibacterial substance was purified by high-pressure liquid chromatography (HPLC). The substance was determined to have a molecular weight of 580.3 *m/z* after analysis by liquid chromatography-mass spectrometry (LC-MS) and was identified as bacillaene (C_34_H_48_N_2_O_6_) by comparison with the standard compounds in the METLIN database (Fig. 6C).

The effect of bacillaene on cell morphology of R. solanacearum was observed by transmission electron microscopy. Bacillaene changed the cell morphology and destroyed the cytoplasmic uniformity of R. solanacearum (Fig. 6D). In the control, R. solanacearum cells were intact, and cytoplasm components were uniformly distributed. After treatment with bacillaene for 24 h, cells were partially damaged, cytoplasm components were concentrated locally, and cell morphology changed to irregular shapes, such as oval and clavate. It was hypothesized that bacillaene disrupted the integrity of the R. solanacearum cell wall and cell membrane and disrupted protein synthesis.

### Single strains and consortia of endophytic bacteria controlled bacterial wilt disease.

*Bacillus* and *Paenibacillus* were identified as the keystone microorganisms of the healthy-plant network. Pseudomonas showed antagonistic and plant growth-promoting activities. We chose six endophytic bacterial isolates belonging to *Bacillus*, *Paenibacillus*, and Pseudomonas to control bacterial wilt, including *B. velezensis* E9, *B. siamensis* E59, *B. amyloliquefaciens* E60, Paenibacillus polymyxa E29, Pseudomonas poae E7, and *P. simiae* E18. Six endophytic bacterial isolates showed high antagonistic and plant growth-promoting activities. The control effect of endophytic bacteria on bacterial wilt was detected by inoculating bacterial fermentations into the tobacco plant rhizosphere. In field experiments, six endophytic bacterial fermentations cultivated in liquid medium significantly (*P < *0.01) decreased disease incidence and the disease severity index (Table 3). The disease incidence in plants treated with *B. velezensis* E9, *B. siamensis* E59, *B. amyloliquefaciens* E60, *P. polymyxa* E29, *P. poae* E7, and *P. simiae* E18 decreased by 64.8%, 55.2%, 68.3%, 79.3%, 64.8%, and 89.6%, respectively. Biocontrol efficacies of endophytic bacteria were 55.3% to 89.6%. *P. simiae* E18 showed the highest biocontrol efficacy (89.6%), followed by *P. polymyxa* E29 (79.3%), *B. amyloliquefaciens* E60 (68.2%), *B. velezensis* E9 (64.7%), and *P. poae* E7 (64.7%) (Table 3).

**TABLE 3:** Biocontrol efficacy of endophytic bacteria consortia on bacterial wilt disease^*a*^

Treatment	Disease incidence (%)	Disease severity index	Biocontrol efficacy (%)
Control	4.89 ± 0.76 a	0.54 ± 0.10 a	
Bacillus velezensis E9	1.72 ± 0.75 bc	0.19 ± 0.06 b	64.71 ± 15.28 bc
Bacillus siamensis E59	2.19 ± 0.24 b	0.24 ± 0.03 b	55.26 ± 4.84 c
Bacillus amyloliquefaciens E60	1.55 ± 0.52 bc	0.17 ± 0.06 b	68.18 ± 10.61 b
Paenibacillus polymyxa E29	1.01 ± 0.0 bc	0.11 ± 0.0 b	79.32 ± 0.0 a
Pseudomonas poae E7	1.72 ± 0.57 bc	0.19 ± 0.06 b	64.71 ± 11.76 bc
Pseudomonas simiae E18	0.51 ± 0.44 c	0.06 ± 0.05 b	89.58 ± 9.02 a
Consortium I (E7, E9, E29)	0.99 ± 0.34 bc	0.11 ± 0.04 b	79.69 ± 7.03 a
Consortium II (E9, E29, E60)	0.60 ± 0.0 bc	0.07 ± 0.0 b	87.82 ± 0.0 a

a Data are averages with standard deviations from triplicate experiments. Different letters in the same column represent significant (*P *< 0.05) differences among treatments.

Consortium I contained *B. velezensis* E9, *P. polymyxa* E29, and *P. poae* E7. Consortium II contained *B. velezensis* E9, *B. amyloliquefaciens* E60, and *P. polymyxa* E29. Application of synthetic microbial consortia significantly reduced the prevalence of bacterial wilt disease. Biocontrol efficacies of consortium I and consortium II were 79.7% and 87.8%. Synthetic microbial consortia showed higher biocontrol efficacy than all single strains, suggesting synergistic actions among different endophytic bacteria.

### The synthetic microbial consortium regulated the endophytic microbiome and improved plant resistance against the pathogen.

The endophytic microbiome and network of tobacco plants treated with consortia were analyzed. The endophytic microbiome and network structure seen with consortium I treatment (R group) changed obviously compared to the control group (C group). Application of consortium I reduced the abundance of the pathogen compared to the control. The relative abundance of R. solanacearum after consortium I treatment (3.2%) was significantly lower than in the control (9.9%). The microbial network in the group receiving consortium treatment had higher connectivity (6.500) and shorter geodesic distance (3.940) than the network of the control group (connectivity, 5.774; geodesic distance, 4.595) (Table 4), indicating that application of the consortium shifted the endophytic network to a more complex network. The microbial network seen with consortium treatment had more negative links (95 links) than the control group (38 links), indicating that competition and antagonism interactions among microorganisms increased in the consortium treatment. It was hypothesized that application of the consortium enabled antagonistic bacteria to engage in negative interactions with the pathogen by inhibiting its growth or competing for nutrients and space.

**TABLE 4:** Microbial network structure of consortium and control treatments

Condition	Empirical networks^*a*^						Random networks (avg ± SD)		
	*S_t_^b^*	Network size (no. of nodes)	Avg connectivity	Avg geodesic distance	Avg clustering coefficient	Modularity (no. of modules)	Geodesic distance	Clustering coefficient	Modularity
Control	0.84	133	5.774	4.595	0.302	0.485 (13)	2.995 ± 0.056	0.113 ± 0.015	0.335 ± 0.008
Consortium	0.80	136	6.500	3.940	0.284	0.480 (6)	2.849 ± 0.034	0.098 ± 0.013	0.321 ± 0.008

a *S_t_^b^*, similarity threshold.

**FIG 7:**
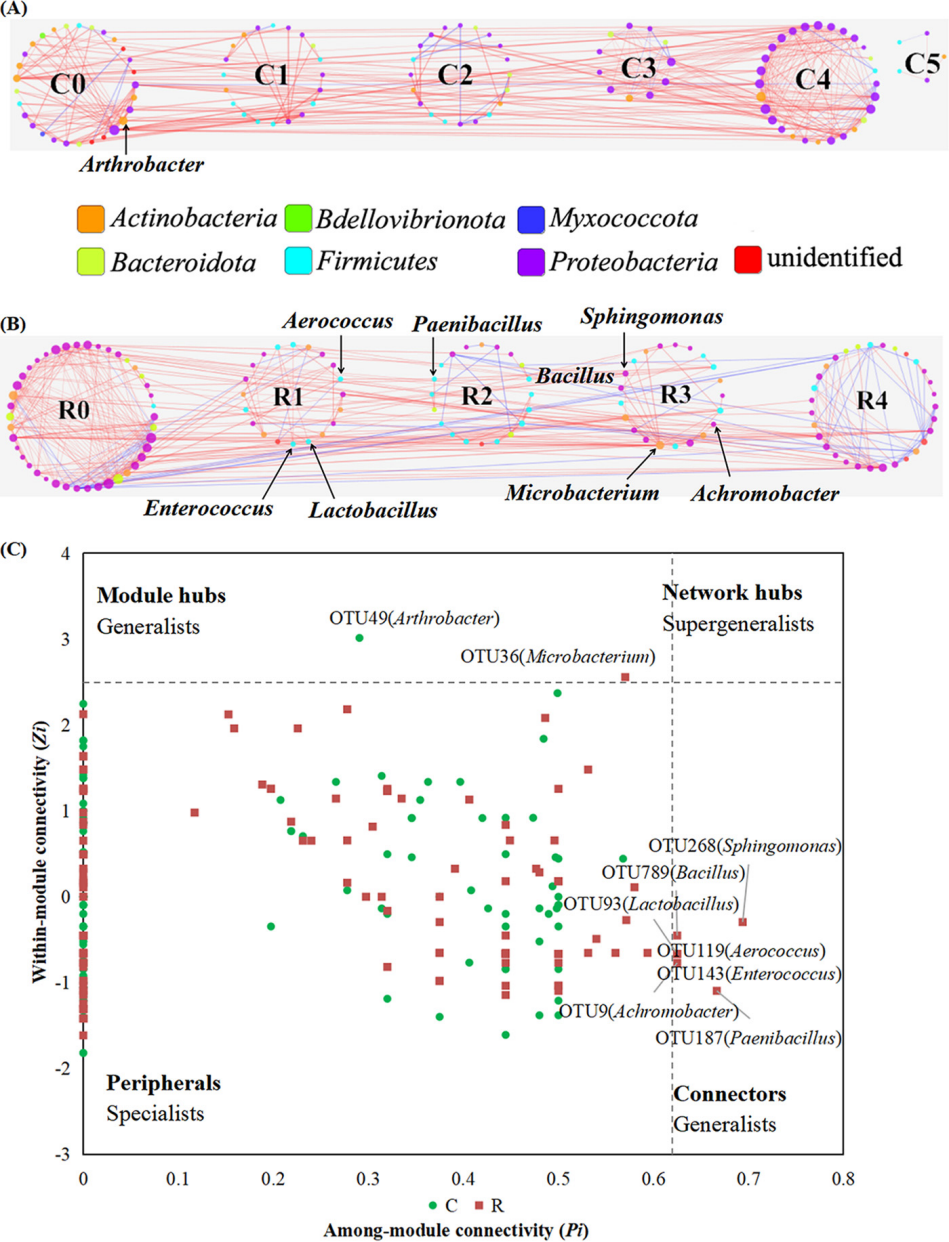
Synthetic microbial consortium regulated endophytic microbial network. (A) Network of the control group. (B) Network of the consortium treatment group. Nodes of different colors belong to different bacterial phyla. Blue edges represent negative interactions between nodes, and red edges represent positive interactions. Modules with five or more nodes are included. Node size is proportionate to the node degree (connectivity of nodes). Edge width is proportionate to the edge betweenness (strength of the correlation). Key microorganisms and module numbers are indicated. (C) Topological roles of nodes. Generalists are labeled with OTU number and phylogenetic relationship. C, control group; R, consortium treatment group.

The keystone microorganism of the consortium treatment was analyzed based on within-module connectivity and connectivity among modules. The network of the consortium treatment had more keystone microorganisms than the control network. One key microorganism, OTU49 (*Arthrobacter*), was found in control network (Fig. 7A and C). Eight keystone microorganisms were found in the consortium treatment network, including OTU9 (*Achromobacter*), OTU36 (*Microbacterium*), OTU93 (*Lactobacillus*), OTU119 (*Aerococcus*), OTU143 (*Enterococcus*), OTU187 (*Paenibacillus*), OTU268 (*Sphingomonas*), and OTU789 (*Bacillus*) (Fig. 7B and C). OTU187 and OTU789 were closely related to *B. velezensis* E9 and *P. polymyxa* E29, which were applied in the field, suggesting that members of consortium applied became keystone microorganisms that could inhibit the pathogen. This result suggested that the synthetic microbial consortium shifted the endophytic microbiome and network to one that effectively suppressed bacterial wilt disease. Endophytic bacteria played important roles in affecting and regulating the endophytic microbiome and effectively controlled infection with R. solanacearum.

### Endophytic bacteria reproduced within plants and promoted plant growth.

Colonization of plants by endophytic bacteria was detected by inoculating bacteria into rhizosphere soil and isolating bacteria from Arabidopsis thaliana Col-0 seedlings. As time goes on, the cell numbers of endophytic bacteria alive in plants gradually increased after inoculation (Fig. S5A). Cell numbers of *P. polymyxa* E29 rose the fastest and reached the highest level (1.15 × 10^7^ CFU g leaf^−1^) at day 28. Cell numbers of *P. poae* E7 and *B. velezensis* E9 reached ~4 × 10^6^ CFU g leaf^−1^ at day 28. Endophytic bacteria colonized stably and reproduced well within plants, which allowed them to play beneficial roles in the plants. For consortium I treatment, cell numbers of bacteria rose smoothly at first and then quickly increased, indicating that the consortium adapted well to the environment inside the plant.

The effect of endophytic bacteria on plant growth was investigated. Endophytic bacteria promoted plant growth (Fig. S5B to D). Fresh weight and dry weight of plants in all treatment groups were significantly (*P < *0.05) higher than those of control plants. *P. polymyxa* E29 showed the best growth-promoting effect, followed by consortium I and *B. velezensis* E9 treatments. Applications of *P. polymyxa* E29, consortium I, and *B. velezensis* E9 resulted in 104.5%, 81.1%, and 77.9% increases in plant biomass, respectively.

### Endophytic bacteria induce systemic resistance in plants.

Endophytic bacteria were inoculated into the plant rhizosphere, and the expression of genes involved in plant resistance was detected by reverse transcription-PCR (RT-PCR). *PR1*, *COI1*, and *ETR1* are defense-related genes involved in salicylic acid (SA), jasmonic acid (JA), and ethylene (ET) pathways, respectively (19). The transcriptional level of *PR1* was decreased in plants treated with endophytic bacteria except for an increase in plants treated with *B. velezensis* E9 at 48 h. This result suggested that endophytic bacteria did not induce defense-related genes of the SA pathway (Fig. S5E). Transcriptional levels of *COI1* showed no significant difference among all treatments at 12 h (Fig. S5F). In plants treated with *B. velezensis* E9, the transcriptional level of *COI1* was 2.2-fold higher than that in the untreated (control) plants at 24 h. In plants treated with *P. polymyxa* E29, the transcriptional level of *COI1* was 1.1-fold higher than that in control plants at 48 h. In plants treated with *P. polymyxa* E29, the transcriptional level of *ETR1* was 1-fold higher than that in the control plants at 48 h (Fig. S5G). These results suggested that *P. polymyxa* E29 induced systemic resistance that was depended on the JA and ET pathways and *B. velezensis* E9 induced systemic resistance that was depended on JA pathway.

Leaves were assessed by staining for production of reactive oxygen species (ROS). Three treatments with endophytic bacteria induced the generation of ROS at 12 h after inoculation (Fig. S5H). At 24 h, the ROS burst was more obvious in plants treated with *B. velezensis* E9 or *P. polymyxa* E29. In summary, *B. velezensis* E9 and *P. polymyxa* E29 induced systemic resistance against the pathogen by enhancing transcription of marker genes of the JA/ET signaling pathways and inducing an ROS burst.

## DISCUSSION

Endophytes live symbiotically with plants and in turn help the plants in a number of ways. In the current study, root endophytic bacteria in healthy and R. solanacearum-infected tobacco plants were analyzed. The endophytic root microbiome and network structure of the diseased plants were distinct from those of healthy plants. Plants with lower species richness in the endophytic root microbiome were more vulnerable to infection. Plant-beneficial endophytic bacteria were enriched in healthy plant root. The healthy-plant network was more complex than the diseased-plant network. Highly connected and complex members of the microbiota and antagonistic endophytic bacteria in healthy plants controlled bacterial wilt disuse. Synthetic microbial consortia containing beneficial endophytic bacteria regulated the endophytic root microbiome and attenuated bacterial wilt disease. Endophytic bacteria inhibited the growth of the pathogen R. solanacearum by producing antibiotics (e.g., bacillaene), inducing systemic resistance, and competing for nutrients and space in the plant.

The pathogen R. solanacearum was the most abundant and core species in the diseased-plant microbiome. In the diseased-plant network, the key microorganism OTU1 was closely related to R. solanacearum. Our results verified that R. solanacearum was the core species that affected the endophytic microbiome and plant health. R. solanacearum was negatively correlated with the diazotrophic bacteria *Pelomonas* and Rhizobium rhizogenes, which might assist the plant to grow more healthily (20).

Actinobacteria produce antibiotics, and cyanobacteria are photosynthetic bacteria and nitrogen-fixing bacteria, so enrichments of *Actinobacteria* and *Cyanobacteria* in healthy plants might inhibit infection and promote plant growth. Relative abundances of plant-beneficial endophytic bacteria decreased in diseased plants compared to healthy plants. *Bacillus*, *Bradyrhizobium*, *Rhizobium*, and *Streptomyces* are often considered plant-beneficial bacteria, indicating that beneficial bacteria were enriched in healthy plants. In particular, *Streptomyces* and *Bacillus* species produce many kinds of antibiotics. *Bacillus* made up a lower proportion (0.08%) of the diseased-plant microbiome and a higher proportion (0.56%) in the healthy-plant microbiome. Strong antagonists belonging to *Bacillus*, Pseudomonas and *Microbacterium* were isolated, and these might be able to be applied as biocontrol agents against bacterial wilt disease. Four endophytic *Bacillus* isolates (i.e., *B. velezensis* E9, *B. velezensis* E10, *B. siamensis* E59, and *B. amyloliquefaciens* E60) exhibited activity against R. solanacearum. The key microorganism OTU399 in the healthy-plant network is closely related to *Bacillus*. Our results indicate that *Bacillus* was the important endophytic bacterium that protected plants against R. solanacearum infection.

It is well known that *Bacillus* species produce numerous antimicrobial compounds (21). Here, it was found that polyene antibiotic bacillaene was the antibacterial substance produced by *B. velezensis* E9. Butcher et al. (22) reported that bacillaene is active against a broad spectrum of bacteria by inhibiting prokaryotic protein synthesis. Here, we report for the first time that bacillaene produced by *Bacillus* inhibited growth of the plant pathogen R. solanacearum. Disruption of bacillaene synthesis genes (i.e., *acpK*, *baeD*, and *pksG*) decreased the antibacterial activity of E9. Complemented (c-Δ*sfp*, c-Δ*pksG*, and c-Δ*acpK*) strains exhibited partly restored antibacterial activity. In complemented strains and overexpressing strains, genes (i.e., *sfp*, *pksG*, and *acpK*) were introduced in the plasmid T2 vector and transferred into mutants or the wild-type strain. Expression of genes on the plasmid may have been different from expression of genes in the genome of E9, which might be regulated by regulatory proteins (e.g., transcription factor). Therefore, the antibacterial activity of complemented strains could not be completely restored, and overexpressing strains did not show a higher antibacterial activity than the wild-type strain.

Some of the enriched endophytic bacteria and biomarkers in healthy plants were potentially plant-beneficial microbes. For example, *Alcaligenes*, *Amycolatopsis*, *Burkholderia*, *Chryseobacterium*, *Lechevalieria*, Pseudomonas, *Streptomyces*, and *Variovorax* species have been shown to have activities against phytopathogens by producing antibiotics, volatile organic compounds, and other antimicrobial substances (23–29). Pseudomonas and *Streptomyces* exert strong bacteriostatic effects on R. solanacearum and inhibit its virulence traits (25, 26). The biocontrol efficacies of Pseudomonas poae E7 (64.7%) and *P. simiae* E18 (89.6%) (Table 3) were higher than those of Pseudomonas brassicacearum J12 (45.5%) reported by Zhou et al. (25). Previous studies have shown that *Bosea*, *Bradyrhizobium*, *Ensifer*, *Mesorhizobium*, Mycobacterium, *Rhizobium*, and *Shinella* species are nitrogen-fixing bacteria that supply nitrogen for plant growth (30–32). As potential plant growth-promoting bacteria, *Brevundimonas*, Ensifer adhaerens, *Mesorhizobium*, *Streptomyces*, *Rhizobium*, and *Variovorax* increase the yield, resistance, and nutritional contents of crops by fixing nitrogen, solubilizing phosphate, and producing ammonia, IAA, siderophores, and 1-aminocyclopropane-1-carboxylic acid deaminase (33–35).

Plant-beneficial microbes enriched in the healthy-plant microbiome might promote plant growth and suppress infection by R. solanacearum. Many plant growth-promoting endophytic bacteria were cultured from healthy plants. Kluyvera intermedia E5 (76.50 mg/L), Pseudomonas extremorientalis E26 (70.18 mg/L), Pseudomonas antarctica E22 (33.98 mg/L) and Streptomyces thermophilus E28 (19.21 mg/L) produced IAA. This was first study to report that *K. intermedia*, *P. antarctica*, *P. extremorientalis*, and S. thermophilus could produce IAA. This was first study to report that *M. maritypicum*, *P. extremorientalis*, *P. poae*, and *P. simiae* possessed antagonistic activity against R. solanacearum. Although some isolates from the microbiome of diseased plants possessed antibacterial activities, produced IAA or siderophores, and formed biofilms, microbiome isolates from healthy plants (18 strains) and less diseased plants (6 strains) possessed antibacterial activities. It was found that the abundances of beneficial microbes (e.g., *Actinobacteria*, *Cyanobacteria*, *Bacillus*, *Bradyrhizobium*, *Rhizobium*, and *Streptomyces*) in the healthy-plant microbiome were higher than in the diseased-plant microbiome (Fig. 2). A higher abundance of beneficial bacteria in a healthy microbiome could control bacterial wilt disease in tobacco.

Endophytic bacteria enriched in diseased plants require more extensive investigation. Johnke et al. (36) reported that *Bdellovibrio* species act as drivers of microbial alpha diversity and are candidates for the restoration of microbiomes and prevention of dysbiosis. Ye et al. (37) reported that *Corallococcus* controls Fusarium wilt disease by regulating the soil microbial community. Our findings revealed that roots of plants with bacterial wilt disease likely recruited specific bacteria (e.g., *Bdellovibrio* and *Corallococcus*) to regulate the endophytic root microbiome.

The identification of key microorganisms was another focus of our research. *Sphingomonas* simultaneously existed as the key microorganism of healthy-plant (i.e., OTU74) and diseased-plant (i.e., OTU79) networks, indicating that it plays important roles in both networks. Asaf et al. (38) reported that *Sphingomonas* species improve plant growth under stress conditions. Except for *Sphingomonas*, most of the keystone microorganisms in the two networks belonged to different genera and families. In the diseased-plant network, keystone microorganisms contained pathogenic microorganisms (e.g., *Rickettsiaceae* and *Ralstonia*). R. solanacearum causes bacterial wilt disease that is severe in diseased fields. Williams et al. (39) reported that *Bacteriovorax* species are predators that prey on many Gram-negative bacteria. It was speculated that the key microorganism *Bacteriovorax* might shape the diseased-plant microbiome.

The healthy-plant network contained more keystone microorganisms than the diseased-plant network. Species of *Paenibacillus* (e.g., *P. polymyxa* E29) and *Bacillus* (e.g., *B. velezensis* E9) promoted plant growth and offered protection against phytopathogens such as R. solanacearum, which was consistent with previous findings (40, 41). Although there were a few pathogenic bacteria in healthy tobacco root, the antagonism between species allowed plants to grow healthily. *Bacillus* species have been widely exploited as microbial biopesticides and represent about half of the commercially bacterial biocontrol products (40). Expósito et al. (42) reported that *Lysobacter* species produce lytic enzymes and antimicrobial compounds. Unfortunately, Lysobacter soli E30 had no antibacterial activity. More endophytic bacteria might be isolated by changing the cultivation method. Conversely, a lack of antagonistic bacteria as keystone microorganisms in the network of diseased plants prevented the plants from resisting R. solanacearum infection. *Pelomonas* is reported to degrade hemicellulose (43). Kumar and Gera (44) found that *Brevundimonas* has plant growth-promoting activities. Macey et al. (45) reported that *Methylovorus* is a methylotroph which participates in cycling of one-carbon compounds. Plant-beneficial keystone microorganisms (e.g., *Bacillus*, *Brevundimonas*, *Lysobacter*, *Methylovorus*, *Paenibacillus*, and *Pelomonas*) in healthy-plant networks might improve plant growth and health by directly stimulating growth, protecting plants from phytopathogen infection, and participating in nutrient cycling.

In the present study, some endophytic bacteria negatively interacted with R. solanacearum. Among them, *R. rhizogenes* fixes nitrogen and is a suitable candidate as a biofertilizer, as reported previously (46). However, Rhizobium tropici E57 could not grow in nitrogen-free medium, and the nitrogenase gene *nifH* was not amplified from the genome of E57. Other endophytic bacteria have nitrogen-fixation potential will be isolated from more tobacco plant roots in future study. Macey et al. (45) reported that *Methylophilaceae* are methylotrophs. Leung et al. (43) reported that *Pelomonas* degrades hemicellulose. *Rhizobium*, *Methylophilaceae*, and *Pelomonas* participate in carbon and nitrogen cycling. Improvement of nutrient cycling in plants seemed to promote plant health and indirectly helped in controlling disease. Some endophytic bacteria positively interacted with R. solanacearum. Among them, six endophytic bacteria (i.e., *Blastococcus*, *Burkholderia*, *Chryseobacterium*, *Lechevalieria*, *L. shinshuensis*, and *Paenibacillus*) have been shown to produce antimicrobial metabolites (23, 29, 34, 41, 47, 48). Paenibacillus polymyxa E29 inhibited growth of R. solanacearum and produced IAA (41.47 mg/L). Three organisms (i.e., *Asticcacaulis*, *L. shinshuensis*, and *Myroides*) exhibit plant growth-promoting attributes and are used as bio-inoculants to enhance plant growth (49, 50); Leung et al. (43) reported that *Pelomonas* degrades hemicellulose. Williams et al. (39) reported that *Bacteriovorax* preys on Gram-negative bacteria. Our results indicate that R. solanacearum invasion enhanced growth of endophytic bacteria with antimicrobial and plant growth-promoting activities and stimulated specific endophytic bacteria for regulating the endophytic microbiome and improving plant health.

Afzal et al. (51) reported that the main mechanisms by which endophytic bacteria provide their protective effects to plants include the induction of natural defenses in the host (induced systemic resistance [ISR]), competition with pathogens for space and nutrients in the plant, and production of antimicrobials. In the present study, plant-beneficial endophytic bacteria promoted plant growth (e.g., *B. velezensis* E9, *P. poae* E7, and *P. polymyxa* E29) and induced systemic resistance of plants. Jinal and Amaresan (52) reported that ISR induction is a promising strategy to control wilt-causing pathogens such as R. solanacearum and Fusarium oxysporum. Plant-beneficial endophytic bacterial isolates had potential applications in biofertilizers and biological control of bacterial wilt disease. Healthy plants resisted bacterial wilt disease because their roots contained not only antagonistic endophytic bacteria but also endophytic bacteria with plant-beneficial activities that synergistically promoted plant growth and health. Therefore, healthy plants had a better ability to prevent and suppress bacterial wilt disease than diseased plants. In addition, plant-beneficial endophytic bacterial isolates were closely related to keystone microorganisms (e.g., *Bacillus*, *Lysobacter*, and *Paenibacillus*) or to species enriched in the healthy-plant microbiome (e.g., Pseudomonas and *Streptomyces*). Our results indicated that culture-dependent method validated the claims of high-throughput sequencing and network analysis.

Previous studies have shown that application of a single antagonist is not enough to combat bacterial wilt disease (7). Sarma et al. (53) reported that a consortium of biological control agents improves efficacy, stability, and uniformity of microbes involved in plant disease control. In this study, it was also found that antagonistic and beneficial endophytic bacteria could be applied together to plants. Synthetic microbial consortia resisted infection better than a single bacterium. The complementary abilities of different endophytic bacteria possibly accounted for the better protective effect of the consortium.

In conclusion, the endophytic root microbiome contributed to plant growth and health. The endophytic root microbiomes and network structures were different between healthy and diseased plants. The keystone microorganisms of the healthy-plant microbiome were plant-beneficial and antagonistic endophytic bacteria. Endophytic bacteria inhibited infection by producing antibiotics (e.g., bacillaene), inducing systemic resistance, and competing with the pathogen for nutrients (e.g., iron) and ecological niches. Ethylene- and jasmonate-regulated defense priming was induced in the presence of endophytic bacteria. Some plant-beneficial endophytic bacteria were found in diseased plant root, which might fight against pathogens. The whole endophytic root microbiome, rather than a single species, was the deciding factor for plant health. Synthetic microbial consortia controlled disease better than single endophytic bacteria. Different plant-beneficial endophytic bacteria worked together to control bacterial wilt disease.

## MATERIALS AND METHODS

### Collection of root samples and investigation of disease severity index.

Ten root samples of bacterial wilt-diseased Nicotiana tabacum cultivar Yunyan 87 (ED) and 10 root samples of healthy plants (EH) from fields situated at Lichuan City were collected. Healthy plants were collected from fields that did not develop disease all year round (incidence rate = 0%). Diseased plants were collected from fields where serious diseases were recorded (incidence rate > 70%, with 1.3 × 10^4^ CFU of R. solanacearum per g soil). For each sample, roots of 15 plants were randomly collected from each field, and then mixed together as a composite sample. The disease severity index (DSI) was recorded according to a previous study (54). The DSI of the diseased-plant group (54.04) was significantly (*P < *0.001) higher than that of the healthy-plant group (0) (Fig. S6).

Roots were surface sterilized with 1% sodium hypochlorite for 5 min and 75% alcohol for 2 min to remove the surface epiphytic bacteria. The sterile water from the last rinse was spread on LB agar plate to check that root surfaces were thoroughly disinfected.

### Analysis of endophytic root microbiomes and microbial networks.

Total DNA was extracted from roots using a FastDNA spin kit (MP Biomedicals). The V4 region of 16S rRNA gene was amplified using primer pairs 515F and 806R. UPARSE was used to remove chimeric sequences and generate OTUs with 97% similarity. The taxonomy of reads was annotated using the SILVA database based on the mothur algorithm. Chloroplast- and mitochondrion-contaminated reads were removed by filtering. Diversity index, microbial community structure, and biomarker species analyses were performed according to previous study (55). Beta diversity on both weighted and unweighted UniFrac analysis was calculated by QIIME software to evaluate differences of samples in species complexity. Principal-coordinate analysis (PCoA) and nonmetric multidimensional scaling (NMDS) analysis were performed with R software to calculate the differences between microbial communities in healthy and diseased plants. Nonparametric analysis of similarities (ANOSIM) was performed using R software to analyze differences in the beta diversity indices between the groups. The statistical significance of differences was assessed using the least-significant-difference (LSD) test with the Holm-Bonferroni adjustment. *P* values of <0.05 were considered statistically significant.

The Molecular Ecological Network Analysis Pipeline (MENAP) was used to construct microbial networks of healthy plants (EH network) and diseased plants (ED network) (55). Only OTUs that were present in eight or more root samples were included in the network analysis. Greedy modularity optimization was used to determine modules. A microbial network was composed of different OTUs (nodes). Positive or negative interactions between OTUs were represented by edges. The keystone microorganisms were identified based on values of within-module connectivity (*Z_i_*) and connectivity among modules (*P_i_*).

### Isolation, identification, and antibacterial activity assay of endophytic bacteria.

Surface-sterilized roots of healthy and diseased tobacco plants that were collected for microbiome analysis were ground with a sterile mortar and pestle. After centrifugation at 3,000 × *g* for 5 min, the supernatant was spread on Trypticase soy broth agar plates and incubated at 37°C for 48 h. Endophytic bacteria with different morphologies were isolated from plates.

Endophytic bacterial isolates were identified through 16S rRNA gene sequencing. 16S rRNA genes were amplified using universal primers 27-F and 1492-R (Table S3). The PCR-amplified products were sequenced. Sequence alignment was performed with MegAlign and Clustal W software. A phylogenetic tree was constructed using MrBayes 3.27 software and then visualized with iTOL.

Antibacterial activities of endophytic bacterial isolates and Bacillus velezensis E9 mutants against R. solanacearum were evaluated through inhibition zone experiments (12). Two hundred microliters of R. solanacearum culture (optical density at 600 nm [OD_600_] ~ 0.6) grown in LB medium was mixed with 20 mL of melted LB agar medium and poured onto plates. Ten microliters of endophytic bacteria (OD_600_ ~ 0.6) grown in LB medium was added to each Oxford cup and incubated at 28°C for 48 h. Antibacterial activity was evaluated by determining the diameter of the inhibition zone.

### Detecting plant growth-promoting traits of endophytic bacteria.

IAA produced by endophytic bacteria was quantitatively detected according to a previous study (12). Endophytic bacterial isolates were incubated in LB medium at 37°C and 200 rpm for 24 h. One milliliter of 2.5 mg/mL tryptophan was added to 4 mL LB medium, 100 μL of bacterial culture was inoculated, and the mixture was incubated at 37°C and 180 rpm for 48 h. Cultures were centrifuged at 12,000 × *g* for 10 min. Supernatant was mixed with an equal volume of Salkowski’s chromogenic reagent, and a reddish color was developed by incubation in the dark for 30 min. The absorbance at 530 nm was measured with a spectrophotometer. The absorbances of different concentrations of IAA were measured to construct a standard curve. IAA concentration was calculated based on the standard curve. LB medium without bacteria was used as the blank. The experiments were performed in three replicates.

For detecting phosphate solubilization activity, endophytic bacterial isolates were inoculated on Monkina medium agar plates and incubated at 28°C for 7 days. The diameters of transparent zones were measured as an indicator of phosphate solubilization activity.

For screening nitrogen-fixing endophytic bacteria, preliminary screening was performed by incubating bacteria in nitrogen-free Ashby medium (10 g L^−1^ sucrose, 0.12 g L^−1^ NaCl, 0.38 g L^−1^ K_2_HPO_4_, 1 g L^−1^ CaCO_3_, 0.1 g L^−1^ MgSO_4_, and 20 g L^−1^ agar [pH 7.2]) at 37°C to select positive strains that grew normally. Rescreening was performed by amplification of the nitrogenase gene *nifH* using nested PCR (56). PCR products were detected by agarose electrophoresis.

Siderophore production was evaluated using chrome-azurol-sulfonate (CAS) agar (6 g L^−1^ casein acid hydrolysate, 6 g L^−1^ succinic acid, 0.12 g L^−1^ MgSO_4_, 0.11 g L^−1^ CaCl_2_, and 20 g L^−1^ agar) plate assay as described previously (12). Filter paper discs containing 5 μL bacterial culture were placed on CAS agar plates and incubated at 37°C for 48 h. The diameters of the bright orange halos were measured.

Biofilm production by endophytic bacteria was detected by the crystal violet staining method (9). Bacteria were incubated in 5 mL LB medium at 37°C and 180 rpm for 6 h. Culture was mixed with 5 mL MSgg medium (100 mM MOPS, 0.5% glycerol, 0.5% glutamate, 5mM potassium phosphate, 50 μg/mL tryptophan, 50 μg/mL phenylalanine, 2 mM MgCl_2_, 50 μM FeCl_3_, 50 μM MnCl_2_, 700 μM CaCl_2_, 1 μM ZnCl_2_, 2 μM thiamine, pH 7.0) and incubated at 28°C for 48 h. Biofilm was stained with 1% crystalline violet for 20 min, washed with phosphate-buffered saline (PBS), and then incubated with 96% ethanol at 28°C for 30 min. The absorbance at 590 nm was measured with a spectrophotometer.

### Constructing mutant strains of Bacillus velezensis E9.

*Bacillus* was identified as one of the keystone microorganisms of the healthy-plant network. Endophytic *B. velezensis* strain E9 showed the highest antibacterial activity. We identified the functional genes involved in antibacterial substance synthesis by constructing mutants of strain E9. Polyketide synthesis genes (*sfp*, *acpK*, *bacE*, *baeB*, *baeC*, *baeD*, and *pksG*) and lipopeptide synthesis genes (*srfA* and *ymfD*) were mutated by the double-crossover homologous recombination method (15). Briefly, two ~500-bp arms homologous to the 5′ and 3′ coding region of the genes were amplified by PCR from strain E9 and ligated by overlapping extension PCR, and the DNA fragments were subcloned into vector T2 (Table S3). The resulting plasmids were transformed into strain E9. Knockout strains were screened out and confirmed by nucleotide sequencing.

Complemented strains were constructed as follows. The corresponding gene and its promoter were amplified from the strain E9 genome and cloned into vector T2. Recombinant plasmids were transformed into knockout strains. Complemented strains were screened in kanamycin-containing LB medium plates. Recombinant plasmids were transformed into wild-type strain E9 to construct overexpressing strains. Antibacterial activities of wild-type, mutant, complemented, and overexpressing strains were detected as described above.

### Purification of bacillaene and analysis of its effect on cell morphology of R. solanacearum.

Bacillaene was extracted and purified according to a previous study (57). *B. velezensis* strain E9 was incubated in production medium (100 mM MOPS [morpholinepropanesulfonic acid], 5 mM KH_2_PO_4_, 0.5% sodium glutamate, 0.5% glycerol, 1 mM MgSO_4_, 100 μM CaCl_2_, 6 μM MnSO_4_, 3 μM FeSO_4_ [pH 7]) at 25°C and 200 rpm for 26 h. Culture was centrifuged at 4,000 × *g* for 30 min. Supernatant was extracted with dichloromethane and saturated NaCl solution in turn. The extract was chromatographed on an Agilent 1200 high-pressure liquid chromatograph (HPLC) with a mobile phase containing acetonitrile. Fractions were eluted with 65% acetonitrile and detected at 361 nm. Fractions containing a bacillaene peak were pooled and analyzed by LC-MS. The antibacterial activity of bacillaene was detected as described above.

R. solanacearum was incubated in LB medium containing 10 μg/mL bacillaene for 48 h. Cultures were collected at 24 h and centrifuged at 8,000 × *g* for 5 min. Cell precipitates were negatively stained with sodium phosphotungstate for 30 s. Cell morphology was observed using a 100 kV transmission electron microscope (Hitachi, Japan) (12). R. solanacearum that was not treated with bacillaene was used as the control.

### Detecting the efficacy of single endophytic bacteria and synthetic microbial consortia as biocontrol for tobacco bacterial wilt disease.

Endophytic bacterial isolates were incubated in LB medium at 37°C and 180 rpm for 36 h. In the field biocontrol experiment, 50 mL pathogen culture (1 × 10^7^ CFU/mL) was used to irrigate the rhizosphere soil of each plant at 1 week after transplantation. After 15 and 30 days, 200 mL of single-isolate culture (1 × 10^8^ CFU/mL) was used to irrigate each tobacco plant. Sixty plants were treated in each plot (6 by 6 m). Three plots were set up for each treatment. Irrigation with an equal volume of water was used as the control.

For constructing synthetic microbial consortia, mixed-culture fermentations of different endophytic bacteria were performed. Three endophytic bacterial isolates were simultaneously inoculated into LB medium with equal amounts of cells and incubated at 37°C and 180 rpm for 36 h. Two hundred milliliters of mixed fermentation broth (1 × 10^8^ CFU/mL) was used to irrigate each plant. Consortium I contained *B. velezensis* E9, *P. polymyxa* E29, and *P. poae* E7 (6:1:2 ratio). Consortium II contained *B. velezensis* E9, *B. amyloliquefaciens* E60, and *P. polymyxa* E29 (5:3:2 ratio). The mixing ratio of strains in the synthetic bacteria was determined by plate dilution and a counting method. Fermentation broth was diluted and spread on LB agar plates. After incubation at 37°C for 48 h, the colonies of the different strains were counted.

After application of microbial agents for 30 days, root samples were collected from the control group and the consortium I treatment group. The endophytic root microbiomes and networks were analyzed to reveal the effect of the consortium on the microbial community. The disease severity index and biocontrol efficacy of each treatment were investigated (58).

### Cocultivation of endophytic bacteria and R. solanacearum.

We adopted a cocultivation method to quantify the interactions between R. solanacearum and the keystone microorganisms of endophytic network, and interactions between endophytic bacteria and pathogen in the subnetwork of R. solanacearum. Initial density of all monocultures was 10^5^ cells per milliliter. The cocultures were inoculated with half of the initial cell density of each species. After culture in LB medium at 28°C for 24 h, cells were spread on LB agar plates. Colonies were identified and counted according to the colony morphology of each species. The type and intensity of promotion and suppression were calculated according to a previous study (59). The type of pairwise interaction between two species was determined by comparing the sum of the endpoint of monoculture productivity of the species with the end productivity of the two-species coculture. In order to characterize directionality of pairwise interactions, we compared the end productivity of each species in two-species cocultures with their end productivities in monocultures. We also calculated the mean intensity of facilitation (MIF) using the following formula: MIF*_ij_* = 0.5 × [log(CP*_i_*_/_MP*_i_*) + log(CP*_j_*_/_MP*_j_*)], where CP*_i_* and CP*_j_* represent the end productivity of each species in two-species cocultures and MP*_i_* and MP*_j_* represent the end productivity in monoculture. The two-species interaction was defined as facilitative when the MIF was >0, antagonistic when the MIF was <0, and neutral when the MIF was 0.

### Detecting plant growth-promoting effects of endophytic bacteria.

We investigated the plant growth-promoting effect of single endophytic bacteria and consortium I. The endophytic bacterial strains E9, E29, and E7 were first induced to express resistance to rifampicin (40 μg/mL). The rifampicin-resistant strain was incubated in LB medium at 37°C and 180 rpm for 24 h. Consortium I was constructed by mixed-culture fermentations as described above. Two weeks after transplantation, 5 mL bacterial culture (1 × 10^8^ CFU/mL) per seedling was used to irrigate Arabidopsis thaliana Col-0. An equal volume of PBS buffer was applied to plants as a blank control. Fifteen plants were treated in each treatment group, with three replications per group.

Cell numbers of endophytic bacteria alive in plants were investigated by the agar plate dilution method on days 0, 14, and 28 after inoculation of bacteria. Leaves were surface sterilized and ground with a sterile mortar and pestle. Supernatant was serially diluted and spread on LB agar plates supplemented with 40 μg/mL rifampicin. After incubation at 37°C for 48 h, bacterial colonies were counted. The fresh weight and dry weight of plants were measured on day 30.

### Detecting induced systemic resistance mediated by endophytic bacteria.

*A. thaliana* plants were irrigated with cultures of *P. poae* E7, *B. velezensis* E9, and *P. polymyxa* E29 as described above. Irrigation with an equal volume of PBS buffer was used as a blank control. Leaf samples were collected at 12 h, 24 h, and 48 h after irrigation treatment. Total RNA was extracted using the TRIzol reagent. cDNA was synthesized using the RNA sample as the template and using an oligo(dT)_18_ primer. Transcriptional levels of *PR1*, *COI1*, and *ETR1* were determined by real-time fluorescence quantitative PCR. The beta-tubulin-encoding gene *beta-Tub* was amplified as a reference gene (Table S4).

ROS production was detected as described previously (60). Leaves were stained with 3′,3-diaminobenzidine (DAB) dye at 25°C for 4 h and treated with anhydrous ethanol at 90°C for 15 min. Leaves were observed under a microscope to detect red-brown deposits.

### Data availability.

Bacterial 16S rRNA sequence data are available at the U.S. National Center for Biotechnology Information (NCBI Sequence Read Archive [SRA], accession number PRJNA800636).

## References

[1] Peeters N, Guidot A, Vailleau F, Valls M. 2013. *Ralstonia solanacearum*, a widespread bacterial plant pathogen in the post-genomic era. Mol Plant Pathol 14:651–662. doi:10.1111/mpp.12038. 23718203 PMC6638647

[2] Li S, Liu Y, Wang J, Yang L, Zhang S, Xu C, Ding W. 2017. Soil acidification aggravates the occurrence of bacterial wilt in south China. Front Microbiol 8:703. doi:10.3389/fmicb.2017.00703. 28487678 PMC5403937

[3] Jiang G, Wei Z, Xu J, Chen H, Zhang Y, She X, Macho AP, Ding W, Liao B. 2017. Bacterial wilt in China: history, current status, and future perspectives. Front Plant Sci 8:1549. doi:10.3389/fpls.2017.01549. 28955350 PMC5601990

[4] Barberán A, Bates ST, Casamayor EO, Fierer N. 2012. Using network analysis to explore co-occurrence patterns in soil microbial communities. ISME J 6:343–351. doi:10.1038/ismej.2011.119. 21900968 PMC3260507

[5] Wei Z, Gu Y, Friman VP, Kowalchuk GA, Xu Y, Shen Q, Jousset A. 2019. Initial soil microbiome composition and functioning predetermine future plant health. Sci Adv 5:eaaw0759. doi:10.1126/sciadv.aaw0759. 31579818 PMC6760924

[6] Mousa WK, Shearer C, Limay-Rios V, Ettinger CL, Eisen JA, Raizada MN. 2016. Root-hair endophyte stacking in finger millet creates a physicochemical barrier to trap the fungal pathogen *Fusarium graminearum*. Nat Microbiol 1:16167. doi:10.1038/nmicrobiol.2016.167. 27669453

[7] Xu XM, Jeffries P, Pautasso M, Jeger MJ. 2011. Combined use of biocontrol agents to manage plant diseases in theory and practice. Phytopathology 101:1024–1031. doi:10.1094/PHYTO-08-10-0216. 21554184

[8] Zhang L, Wang D, Hu Q, Dai X, Xie Y, Li Q, Liu H, Guo J. 2019. Consortium of plant growth-promoting rhizobacteria strains suppresses sweet pepper disease by altering the rhizospheric microbiota. Front Microbiol 10:1668. doi:10.3389/fmicb.2019.01668. 31396185 PMC6664061

[9] Berendsen RL, Vismans G, Yu K, Song Y, de Jonge R, Burgman WP, Burmølle M, Herschend J, Bakker PAHM, Pieterse CMJ. 2018. Disease-induced assemblage of a plant-beneficial bacterial consortium. ISME J 12:1496–1507. doi:10.1038/s41396-018-0093-1. 29520025 PMC5956071

[10] Qiu JP, Huang YX, Wang C, Yu YY, Ke HJ, Guo JH. 2014. Effects of bacterial consortium EG03 on control of pepper bacterial wilt and rhizosphere microbial community characteristics in fields. Ying Yong Sheng Tai Xue Bao 25:1468–1474. 25129950

[11] Olesen JM, Bascompte J, Dupont YL, Jordano P. 2007. The modularity of pollination networks. Proc Natl Acad Sci USA 104:19891–19896. doi:10.1073/pnas.0706375104. 18056808 PMC2148393

[12] Yuan W, Ruan S, Qi G, Wang R, Zhao X. 2022. Plant growth-promoting and antibacterial activities of cultivable bacteria alive in tobacco field against *Ralstonia solanacearum*. Environ Microbiol 24:1411–1429. doi:10.1111/1462-2920.15868. 35112429

[13] Erega A, Stefanic P, Dogsa I, Danevčič T, Simunovic K, Klančnik A, Možina SS, Mulec IM. 2021. Bacillaene mediates the inhibitory effect of *Bacillus subtilis* on *Campylobacter jejuni* biofilms. Appl Environ Microbiol 87:e02955-20. doi:10.1128/AEM.02955-20. 33837012 PMC8174767

[14] Islam T, Rabbee MF, Choi J, Baek KH. 2022. Biosynthesis, molecular regulation, and application of bacilysin produced by *Bacillus* species. Metabolites 12:397. doi:10.3390/metabo12050397. 35629901 PMC9147277

[15] Chen B, Wen J, Zhao X, Ding J, Qi G. 2020. Surfactin: a quorum-sensing signal molecule to relieve CCR in *Bacillus amyloliquefaciens*. Front Microbiol 11:631. doi:10.3389/fmicb.2020.00631. 32425896 PMC7203447

[16] Miethke M, Klotz O, Linne U, May JJ, Beckering CL, Marahiel MA. 2006. Ferri-bacillibactin uptake and hydrolysis in *Bacillus subtilis*. Mol Microbiol 61:1413–1427. doi:10.1111/j.1365-2958.2006.05321.x. 16889643

[17] Patel PS, Huang S, Fisher S, Pirnik D, Aklonis C, Dean L, Meyers E, Fernandes P, Mayerl F. 1995. Bacillaene, a novel inhibitor of procaryotic protein synthesis produced by *Bacillus subtilis*: production, taxonomy, isolation, physico-chemical characterization and biological activity. J Antibiot (Tokyo) 48:997–1003. doi:10.7164/antibiotics.48.997. 7592068

[18] Harwood CR, Mouillon J, Pohl S, Arnau J. 2018. Secondary metabolite production and the safety of industrially important members of the *Bacillus subtilis* group. FEMS Microbiol Rev 42:721–738. doi:10.1093/femsre/fuy028. 30053041 PMC6199538

[19] Pieterse CM, Zamioudis C, Berendsen RL, Weller DM, Van Wees SC, Bakker PA. 2014. Induced systemic resistance by beneficial microbes. Annu Rev Phytopathol 52:347–375. doi:10.1146/annurev-phyto-082712-102340. 24906124

[20] Terakado-Tonooka J, Ohwaki Y, Yamakawa H, Tanaka F, Yoneyama T, Fujihara S. 2008. Expressed *nifH* genes of endophytic bacteria detected in field-grown sweet potatoes (*Ipomoea batatas* L.). Microbes Environ 23:89–93. doi:10.1264/jsme2.23.89. 21558693

[21] Chen XH, Koumoutsi A, Scholz R, Borriss R. 2009. More than anticipated—production of antibiotics and other secondary metabolites by *Bacillus amyloliquefaciens* FZB42. J Mol Microbiol Biotechnol 16:14–24. doi:10.1159/000142891. 18957859

[22] Butcher RA, Schroeder FC, Fischbach MA, Straight PD, Kolter R, Walsh CT, Clardy J. 2007. The identification of bacillaene, the product of the PksX megacomplex in *Bacillus subtilis*. Proc Natl Acad Sci USA 104:1506–1509. doi:10.1073/pnas.0610503104. 17234808 PMC1785240

[23] Castanheira N, Dourado AC, Kruz S, Alves PIL, Delgado-Rodríguez AI, Pais I, Semedo J, Scotti-Campos P, Sánchez C, Borges N, Carvalho G, Crespo MTB, Fareleira P. 2016. Plant growth-promoting *Burkholderia* species isolated from annual ryegrass in Portuguese soils. J Appl Microbiol 120:724–739. doi:10.1111/jam.13025. 26671760

[24] Kapley A, Tanksale H, Sagarkar S, Prasad AR, Kumar RA, Sharma N, Qureshi A, Purohit HJ. 2016. Antimicrobial activity of *Alcaligenes* sp. HPC 1271 against multidrug resistant bacteria. Funct Integr Genomics 16:57–65. doi:10.1007/s10142-015-0466-8. 26432787

[25] Zhou T, Chen D, Li C, Sun Q, Li L, Liu F, Shen Q, Shen B. 2012. Isolation and characterization of *Pseudomonas brassicacearum* J12 as an antagonist against *Ralstonia solanacearum* and identification of its antimicrobial components. Microbiol Res 167:388–394. doi:10.1016/j.micres.2012.01.003. 22361469

[26] Wattana-Amorn P, Charoenwongsa W, Williams C, Crump MP, Apichaisataienchote B. 2016. Antibacterial activity of cyclo(L-Pro-L-Tyr) and cyclo(D-Pro-L-Tyr) from *Streptomyces* sp. strain 22–4 against phytopathogenic bacteria. Nat Prod Res 30:1980–1983. doi:10.1080/14786419.2015.1095747. 26469746

[27] Hong CE, Jo SH, Jo IH, Jeong H, Park JM. 2017. Draft genome sequence of the endophytic bacterium *Variovorax paradoxus* KB5, which has antagonistic activity against a phytopathogen, *Pseudomonas syringae* pv. tomato DC3000. Genome Announc 5:e00950-17. doi:10.1128/genomeA.00950-17. 28883145 PMC5589539

[28] Xu X, Han L, Zhao L, Chen X, Miao C, Hu L, Huang X, Chen Y, Li Y. 2019. Echinosporin antibiotics isolated from *Amycolatopsis* strain and their antifungal activity against root-rot pathogens of the *Panax notoginseng*. Folia Microbiol (Praha) 64:171–175. doi:10.1007/s12223-018-0642-z. 30117099

[29] Dahal RH, Chaudhary DK, Kim DU, Pandey RP, Kim J. 2021. *Chryseobacterium antibioticum* sp. nov. with antimicrobial activity against Gram-negative bacteria, isolated from Arctic soil. J Antibiot (Tokyo) 74:115–123. doi:10.1038/s41429-020-00367-1. 32895493

[30] Xu L, Mohamad OA, Ma Y, Zhang Y, Kong Z. 2015. Phylogenetic diversity on housekeeping and symbiotic genes of rhizobial from *Sphaerophysa* in China. World J Microbiol Biotechnol 31:1451–1459. doi:10.1007/s11274-015-1898-y. 26149957

[31] Andrews M, Andrews ME. 2017. Specificity in legume-rhizobia symbioses. Int J Mol Sci 18:705. doi:10.3390/ijms18040705. 28346361 PMC5412291

[32] Rilling JI, Acuña JJ, Sadowsky MJ, Jorquera MA. 2018. Putative nitrogen-fixing bacteria associated with the rhizospheric and root endosphere of wheat plants grown in an Andisol from southern Chile. Front Microbiol 9:2710. doi:10.3389/fmicb.2018.02710. 30524385 PMC6256256

[33] Zhou G, Wang Y, Zhai S, Ge F, Liu Z, Dai Y, Yuan S, Hou J. 2013. Biodegradation of the neonicotinoid insecticide thiamethoxam by the nitrogen-fixing and plant-growth-promoting rhizobacterium *Ensifer adhaerens* strain TMX-23. Appl Microbiol Biotechnol 97:4065–4074. doi:10.1007/s00253-012-4638-3. 23274958

[34] Liaqat F, Eltem R. 2016. Identification and characterization of endophytic bacteria isolated from in vitro cultures of peach and pear rootstocks. 3 Biotech 6:120. doi:10.1007/s13205-016-0442-6. PMC4909027 28330195

[35] Natsagdorj O, Sakamoto H, Santiago DMO, Santiago CD, Orikasa Y, Okazaki K, Ikeda S, Ohwada T. 2019. *Variovorax* sp. has an optimum cell density to fully function as a plant growth promoter. Microorganisms 7:82. doi:10.3390/microorganisms7030082. 30875976 PMC6462933

[36] Johnke J, Fraune S, Bosch TCG, Hentschel U, Schulenburg H. 2020. *Bdellovibrio* and like organisms are predictors of microbiome diversity in distinct host groups. Microb Ecol 79:252–257. doi:10.1007/s00248-019-01395-7. 31187177

[37] Ye X, Li Z, Luo X, Wang W, Li Y, Li R, Zhang B, Qiao Y, Zhou J, Fan J, Wang H, Huang Y, Cao H, Cui Z, Zhang R. 2020. A predatory myxobacterium controls cucumber *Fusarium* wilt by regulating the soil microbial community. Microbiome 8:49. doi:10.1186/s40168-020-00824-x. 32252828 PMC7137222

[38] Asaf S, Numan M, Khan AL, Al-Harrasi A. 2020. *Sphingomonas*: from diversity and genomics to functional role in environmental remediation and plant growth. Crit Rev Biotechnol 40:138–152. doi:10.1080/07388551.2019.1709793. 31906737

[39] Williams HN, Turng BF, Kelley JI. 2009. Survival response of *Bacteriovorax* in surface biofilm versus suspension when stressed by extremes in environmental conditions. Microb Ecol 58:474–484. doi:10.1007/s00248-009-9499-7. 19267151

[40] Favel D. 2005. Commercialization and implementation of biocontrol. Annu Rev Phytopathol 43:337–359. doi:10.1146/annurev.phyto.43.032904.092924. 16078888

[41] Grady EN, MacDonald J, Liu L, Richman A, Yuan ZC. 2016. Current knowledge and perspectives of *Paenibacillus*: a review. Microb Cell Fact 15:203. doi:10.1186/s12934-016-0603-7. 27905924 PMC5134293

[42] Expósito RG, Postma J, Raaijmakers JM, Bruijn ID. 2015. Diversity and activity of *Lysobacter* species from disease suppressive soils. Front Microbiol 6:1243. 26635735 10.3389/fmicb.2015.01243 PMC4644931

[43] Leung HTC, Maas KR, Wilhelm RC, Mohn WW. 2016. Long-term effects of timber harvesting on hemicellulolytic microbial populations in coniferous forest soils. ISME J 10:363–375. doi:10.1038/ismej.2015.118. 26274049 PMC4737928

[44] Kumar V, Gera R. 2014. Isolation of a multi-trait plant growth promoting *Brevundimonas* sp. and its effect on the growth of Bt-cotton. 3 Biotech 4:97–101. doi:10.1007/s13205-013-0126-4. PMC3909574 28324462

[45] Macey MC, Pratscher J, Crombie A, Murrell JC. 2018. Draft genome sequences of obligate methylotrophs *Methylovorus* sp. strain MM2 and *Methylobacillus* sp. strain MM3, isolated from grassland soil. Microbiol Resour Announc 7:e00824-18. doi:10.1128/MRA.00824-18. 30533926 PMC6256518

[46] Ruiz-Díez B, Fajardo S, Puertas-Mejía MA, Felipe MR, Fernández-Pascual M. 2009. Stress tolerance, genetic analysis and symbiotic properties of root-nodulating bacteria isolated from *Mediterranean leguminous* shrubs in Central Spain. Arch Microbiol 191:35–46. doi:10.1007/s00203-008-0426-y. 18784916

[47] Rasuk MC, Ferrer GM, Kurth D, Portero LR, Farías ME, Albarracín VH. 2017. UV-resistant *Actinobacteria* from high-altitude Andean lakes: isolation, characterization and antagonistic activities. Photochem Photobiol 93:865–880. doi:10.1111/php.12759. 28500722

[48] Shan W, Zhou Y, Liu H, Yu X. 2018. Endophytic actinomycetes from tea plants (*Camellia sinensis*): isolation, abundance, antimicrobial, and plant-growth-promoting activities. Biomed Res Int 2018:1470305. 30519568 10.1155/2018/1470305 PMC6241348

[49] Kaur R, Kaur S. 2021. Plant growth-promoting potential of ‘*Myroides gitamensis*’ isolated from virgin soils of Punjab. Arch Microbiol 203:2551–2561. doi:10.1007/s00203-021-02231-8. 33683396

[50] Okazaki K, Tsurumaru H, Hashimoto M, Takahashi H, Okubo T, Ohwada T, Minamisawa K, Ikeda S. 2021. Community analysis-based screening of plant growth-promoting bacteria for sugar beet. Microbes Environ 36:ME20137. 33907063 10.1264/jsme2.ME20137 PMC8209457

[51] Afzal I, Shinwari ZK, Sikandar S, Shahzad S. 2019. Plant beneficial endophytic bacteria: mechanisms, diversity, host range and genetic determinants. Microbiol Res 221:36–49. doi:10.1016/j.micres.2019.02.001. 30825940

[52] Jinal NH, Amaresan N. 2020. Evaluation of biocontrol *Bacillus* species on plant growth promotion and systemic-induced resistant potential against bacterial and fungal wilt-causing pathogens. Arch Microbiol 202:1785–1794. doi:10.1007/s00203-020-01891-2. 32382765

[53] Sarma BK, Yadav SK, Singh S, Singh HB. 2015. Microbial consortium-mediated plant defense against phytopathogens: readdressing for enhancing efficacy. Soil Biol Biochem 87:25–33. doi:10.1016/j.soilbio.2015.04.001.

[54] Yi Y, Liu R, Yin H, Luo K, Liu E, Liu X. 2007. Isolation, identification and field control efficacy of an endophytic strain against tobacco bacterial wilt (*Ralstonia solanacarum*). Ying Yong Sheng Tai Xue Bao 18:554–558. 17552192

[55] Qi G, Ma G, Chen S, Lin C, Zhao X. 2019. Microbial network and soil properties are changed in bacterial wilt-susceptible soil. Appl Environ Microbiol 85:e00162-19. doi:10.1128/AEM.00162-19. 31003986 PMC6581179

[56] Ding Y, Wang J, Liu Y, Chen S. 2005. Isolation and identification of nitrogen‐fixing bacilli from plant rhizospherics in Beijing region. J Appl Microbiol 99:1271–1281. doi:10.1111/j.1365-2672.2005.02738.x. 16238759

[57] Müller S, Strack SN, Hoefler BC, Straight PD, Kearns DB, Kirby JR. 2014. Bacillaene and sporulation protect *Bacillus subtilis* from predation by *Myxococcus xanthus*. Appl Environ Microbiol 80:5603–5610. doi:10.1128/AEM.01621-14. 25002419 PMC4178607

[58] Wang R, Zhang H, Sun L, Qi G, Chen S, Zhao X. 2017. Microbial community composition is related to soil biological and chemical properties and bacterial wilt outbreak. Sci Rep 7:343. doi:10.1038/s41598-017-00472-6. 28336973 PMC5428506

[59] Li M, Wei Z, Wang J, Jousset A, Friman VP, Xu Y, Shen Q, Pommier T. 2019. Facilitation promotes invasions in plant-associated microbial communities. Ecol Lett 22:149–158. doi:10.1111/ele.13177. 30460736

[60] Daudi A, O’Brien JA. 2012. Detection of hydrogen peroxide by DAB staining in *Arabidopsis* leaves. Bio Protoc 2:e263. PMC4932902 27390754

