# Supplementary material for: The Endophytic Root Microbiome Is Different in Healthy and Ralstonia solanacearum-Infected Plants and Is Regulated by a Consortium Containing Beneficial Endophytic Bacteria
Source: Microbiol Spectr. 2022 Dec 14;11(1):e02031-22. doi: 10.1128/spectrum.02031-22 (PMC9927471; doi:10.1128/spectrum.02031-22)

Table S1 Activities of root endophytic bacteria

Strains	Inhibition zone diameter (cm)	IAA concentration (mg/L)	Nitrogen fixation	Siderophore production (cm)[a]	Organic phosphorus solubilization (cm)[b]
Achromobacter marplatensis E54	-	4.56 ± 0.23	+	-	-
Bacillus altitudinis E12	-	12.01 ± 4.51	-	-	-
Bacillus amyloliquefaciens E60	2.45 ± 0.13	12.75 ± 0.27	-	-	-
Bacillus aryabhattai E14	-	43.67 ± 17.09	+	-	-
Bacillus cereus E11	-	19.67 ± 0.94	-	-	-
Bacillus siamensis E59	2.60 ± 0.10	12.88 ± 0.17	-	-	-
Bacillus subtilis E8	-	19.01 ± 3.68	+	0.40 ± 0.00	-
Bacillus velezensis E9	2.33 ± 0.12	14.90 ± 1.52	+	0.40 ± 0.00	0.53 ± 0.12
Bacillus velezensis E10	1.33 ± 0.12	11.03 ± 0.27	-	0.35 ± 0.12	0.80 ± 0.00
Bordetella petrii E6	-	6.94 ± 0.05	-	-	-
Burkholder cepacia E55	-	3.45 ± 0.24	-	-	-
Comamonas terrigena E56	-	8.79 ± 0.48	-	-	-
Corticimicrobacter populi E47	-	61.91 ± 20.47	-	0.18 ± 0.04	-
Corticimicrobacter populi E52	-	45.52 ± 2.65	-	0.20 ± 0.04	-
Delftia lacustris E49	-	23.27 ± 11.17	-	-	-
Empedobacter brevis E33	-	5.61 ± 0.16	-	0.28 ± 0.04	-
Enterobacter kobei E40	-	88.20 ± 16.93	-	-	0.56 ± 0.06
Enterobacter ludwigii E35	-	141.64 ± 57.69	-	0.30 ± 0.10	0.42 ± 0.04
Enterococcus phoeniculicola E39	-	36.13 ± 11.59	-	0.30 ± 0.08	-
Klebsiella pneumoniae E32	-	181.55 ± 30.81	-	-	0.40 ± 0.14
Kluyvera intermedia E2	-	63.08 ± 4.94	-	-	-
Kluyvera intermedia E5	-	76.50 ± 14.72	-	-	-
Lysobacter soli E30	-	13.84 ± 4.05	-	-	-
Microbacterium maritypicum E21	2.13 ± 0.12	17.51 ± 3.20	-	0.20 ± 0.00	0.60 ± 0.00
Microbacterium saperdae E46	-	15.95 ± 10.60	-	-	-
Morganella morganii E31	-	145.03 ± 12.84	-	-	0.26 ± 0.06
Paenibacillus glycanilyticus E58	-	8.33 ± 0.46	-	-	-
Paenibacillus polymyxa E29	0.20 ± 0.00	41.47 ± 24.63	-	-	-
Pantoea agglomerans E51	-	115.04 ± 7.11	-	0.36 ± 0.08	-
Pantoea rodasii E1	-	54.19 ± 1.82	-	-	-
Pseudomonas antarctica E22	0.33 ± 0.12	33.98 ± 1.74	+	-	1.13 ± 0.12
Pseudomonas brassicacearum E16	0.13 ± 0.06	15.86 ± 10.31	-	-	0.67 ± 0.12
Pseudomonas entomophila E37	0.71 ± 0.13	9.42 ± 5.00	-	0.22 ± 0.06	0.46 ± 0.10
Pseudomonas entomophila E41	0.77 ± 0.19	9.59 ± 5.94	-	-	0.32 ± 0.30
Pseudomonas extremorientalis E13	1.93 ± 0.12	6.07 ± 0.63	+	-	-
Pseudomonas extremorientalis E26	-	70.18 ± 5.23	+	-	0.40 ± 0.00
Pseudomonas extremorientalis E27	0.20 ± 0.00	7.54 ± 1.10	-	-	-
Pseudomonas fluorescens E24	0.20 ± 0.00	6.88 ± 0.63	-	-	-
Pseudomonas geniculata E53	-	7.65 ± 6.14	-	-	-

Pseudomonas nitroreducens E34	-	14.27 ± 3.61	-	-	-
Pseudomonas poae E7	1.53 ± 0.12	12.53 ± 3.90	-	0.60 ± 0.00	1.00 ± 0.00
Pseudomonas poae E17	2.20 ± 0.20	15.01 ± 1.55	-	0.40 ± 0.00	0.47 ± 0.12
Pseudomonas putida E3	0.20 ± 0.00	19.51 ± 7.89	-	-	-
Pseudomonas putida E4	0.20 ± 0.00	21.31 ± 2.11	-	-	0.40 ± 0.00
Pseudomonas putida E20	0.20 ± 0.00	14.22 ± 8.18	-	-	0.47 ± 0.12
Pseudomonas putida E23	1.93 ± 0.12	6.01 ± 1.63	-	-	0.60 ± 0.00
Pseudomonas putida E44	0.86 ± 0.06	24.14 ± 7.16	-	-	0.38 ± 0.04
Pseudomonas putida E45	0.76 ± 0.06	12.77 ± 1.73	-	-	0.44 ± 0.14
Pseudomonas simiae E15	0.20 ± 0.00	65.18 ± 4.58	-	-	0.20 ± 0.00
Pseudomonas simiae E18	2.00 ± 0.00	10.43 ± 1.13	+	0.40 ± 0.00	0.80 ± 0.00
Pseudomonas taiwanensis E43	-	6.29 ± 0.84	-	-	0.80 ± 0.04
Pseudomonas trivialis E25	-	154.06 ± 0.63	+	-	0.73 ± 0.12
Raoultella ornithinolytica E48	-	215.23 ± 58.12	-	-	-
Raoultella planticola E36	-	248.61 ± 47.20	-	-	0.38 ± 0.04
Rhizobium tropici E57	-	13.08 ± 0.26	-	-	-
Stenotrophomonas maltophilia E38	-	26.00 ± 14.75	-	-	-
Stenotrophomonas maltophilia E42	-	38.56 ± 9.94	-	-	-
Stenotrophomonas pavanii E50	-	26.51 ± 13.85	-	-	-
Streptomyces thermophilus E28	-	19.21 ± 0.45	-	0.20 ± 0.00	-
Streptomyces vinaceus E19	0.27 ± 0.12	5.82 ± 0.47	+	-	-

[a]Value represents transparent zone diameter on CAS plate.

[b]Value represents transparent zone diameter on Monkina medium agar plate.

+ represents positive activity. - represents no activity.

Table S2 Interactions between *R. solanacearum* and endophytic bacteria isolates

	Effect of *R. solanacearum* on isolate's growth	Effect of isolate on *R. solanacearum* growth	MIF
E1	-0.59	0.23	-0.18
E2	-0.49	0.24	-0.13
E3	0.78	-0.55	0.12
E4	1.89	-0.04	0.93
E5	-0.66	0.00	-0.33
E6	2.83	1.40	2.11
E7	-0.26	-1.36	-0.81
E8	0.49	0.01	0.25
E9	0.50	-0.55	-0.03
E10	-0.50	-0.95	-0.72
E11	-0.52	-0.83	-0.68
E12	-0.45	0.21	-0.12
E13	-0.10	-0.23	-0.16
E14	0.81	0.29	0.55
E15	-0.51	-0.71	-0.61
E16	-0.21	-0.47	-0.34
E17	1.38	-0.69	0.34

E18	0.87	-0.20	0.34
E19	0.60	-0.10	0.25
E20	0.45	-0.70	-0.13
E21	0.92	-0.63	0.15
E22	0.45	-1.01	-0.28
E23	1.21	-0.95	0.13
E24	1.19	-0.15	0.52
E25	0.92	0.02	0.47
E26	0.91	0.01	0.46
E27	-0.26	-0.42	-0.34
E28	0.52	-0.58	-0.03
E29	-0.31	-1.69	-1.00
E30	0.96	0.17	0.56
E31	0.44	0.96	0.70
E32	0.34	0.98	0.66
E33	1.18	1.66	1.42
E34	1.15	0.30	0.72
E35	0.24	-0.10	0.07
E36	0.07	-0.30	-0.11
E37	-0.66	-0.22	-0.44
E38	-0.38	-1.39	-0.89
E39	0.29	-0.26	0.01
E40	0.39	0.94	0.66
E41	-0.20	-0.56	-0.38
E42	2.17	1.10	1.64
E43	0.33	0.02	0.17
E44	0.76	-0.82	-0.03
E45	0.15	0.03	0.09
E46	0.42	-0.66	-0.12
E47	-0.11	-1.24	-0.67
E48	0.42	1.05	0.73
E49	1.17	0.31	0.74
E50	0.34	1.19	0.76
E51	0.59	1.20	0.90
E52	0.69	-0.26	0.21
E53	-0.35	0.27	-0.04
E54	0.20	1.03	0.61
E55	1.68	-0.20	0.74
E56	0.69	0.06	0.37
E57	-1.42	1.86	0.22
E58	0.75	-0.04	0.35
E59	0.02	-0.94	-0.46
E60	0.66	-1.23	-0.28

MIF represent the mean intensity of facilitation of co-cultures.

Table S3 Primers for PCR used in this study

Name of primer	Primer sequence (5'-3')	Purpose
sfp-LF	CGGGATCCTATCTTAAACATGGTG	Amplification of *sfp* gene 5’ coding region
sfp-LR	CTTTTTCTATGCGTCCAGATCCTC	Amplification of *sfp* gene 5’ coding region
sfp-RF	GAGGATCTGGACGCATAGAAAAAG	Amplification of *sfp* gene 3’ coding region
sfp-RR	GCTCTAGACATTCAGGAATCCATC	Amplification of *sfp* gene 3’ coding region
acpk-LF	CGGGATCCTCTGCGACAAAATAAA	Amplification of *acpk* gene 5’ coding region
acpk-LR	GAGGAACTGACATAAAACGGTGAA	Amplification of *acpk* gene 5’ coding region
acpk-RF	TTCACCGTTTTATGTCAGTTCCTC	Amplification of *acpk* gene 3’ coding region
acpk-RR	GCTCTAGACCTGCAGCAGATGAAT	Amplification of *acpk* gene 3’ coding region
bacE-LF	CGGGATCCGCAACACATACAGAGA	Amplification of *bacE* gene 5’ coding region
bacE-LR	CTGAAAGGCTCCCGGTAACGCATA	Amplification of *bacE* gene 5’ coding region
bacE-RF	TATGCGTTACCGGGAGCCTTTCAG	Amplification of *bacE* gene 3’ coding region
bacE-RR	GCTCTAGACATAAAACGCCTGATC	Amplification of *bacE* gene 3’ coding region
baeB-LF	CGGGATCCTTTATGCCATTGTCTT	Amplification of *baeB* gene 5’ coding region
baeB-LR	CCCGGATCTTTTTGAATGGCTGAG	Amplification of *baeB* gene 5’ coding region
baeB-RF	CTCAGCCATTCAAAAAGATCCGGG	Amplification of *baeB* gene 3’ coding region
baeB-RR	GCTCTAGAAGTAAAAAGCTGCGCG	Amplification of *baeB* gene 3’ coding region
baeC-LF	CGGGATCCAGCACGCAATCTAAAG	Amplification of *baeC* gene 5’ coding region
baeC-LR	ATGGCTGAGAAACGGAACGTAAGT	Amplification of *baeC* gene 5’ coding region
baeC-RF	ACTTACGTTCCGTTTCTCAGCCAT	Amplification of *baeC* gene 3’ coding region
baeC-RR	GCTCTAGATCAAAGCCTCCATTCC	Amplification of *baeC* gene 3’ coding region
baeD-LF	CGGGATCCCTGACATTTAAGCGGT	Amplification of *baeD* gene 5’ coding region
baeD-LR	AAAGGAGAACAACAGGCCAAGGCT	Amplification of *baeD* gene 5’ coding region
baeD-RF	AGCCTTGGCCTGTTGTTCTCCTTT	Amplification of *baeD* gene 3’ coding region
baeD-RR	GCTCTAGACAGACGCCCTTCAAAC	Amplification of *baeD* gene 3’ coding region
baeE-LF	CGGGATCCTCATCAGTAAATTCTC	Amplification of *baeE* gene 5’ coding region
baeE-LR	GGAGACTGTAAGCAAGACGAAATG	Amplification of *baeE* gene 5’ coding region
baeE-RF	CATTTCGTCTTGCTTACAGTCTCC	Amplification of *baeE* gene 3’ coding region
baeE-RR	GCTCTAGACATTACGGAAACTTGC	Amplification of *baeE* gene 3’ coding region
pksG-LF	CGGGATCCTTTCTCAGCTCTGTTG	Amplification of *pksG* gene 5’ coding region
pksG-LR	GAGGCGAAGTTCGATAAACGTCCG	Amplification of *pksG* gene 5’ coding region
pksG-RF	CGGACGTTTATCGAACTTCGCCTC	Amplification of *pksG* gene 3’ coding region

pksG_2-RR	GCTCTAGAAAGTTCTGCTTCGGCG	Amplification of *pksG* gene 3’ coding region
T2-F	TTAACGAATTCCTGCAGCCC	Validation of vector transformation
T2-R	TTTTCTACGAGCTCCACCGC	Validation of vector transformation
T2-dan-LR	TTGTTTGCAAGCAGCAGATT	Single exchange validation
T2-dan-RF	CGAAAAACAAGTTAAGGGAT	Single exchange validation
sfp-dan-LF	GCATTTCGGGCTTTCTGTCG	Validation of single exchange of *sfp* gene
sfp-dan-RR	GCCGGCAAAGAGATTGTCGT	Validation of single exchange of *sfp* gene
acpk-dan-LF	GCCTTCGGCAATTAAAAACC	Validation of single exchange of *acpk* gene
acpk-dan-RR	AGCGGCCTCGTAAAAGACCT	Validation of single exchange of *acpk* gene
bacE-dan-LF	ATCTTGGATGATGACGCGAA	Validation of single exchange of *bacE* gene
bacE-dan-RR	CTGATCAAATTCGAACGCGC	Validation of single exchange of *bacE* gene
baeB-dan-LF	ACAAGCTGAAGGCCGGTAAT	Validation of single exchange of *baeB* gene
baeB-dan-RR	AGGGCAATTTACCTTGAGCA	Validation of single exchange of *baeB* gene
baeC-dan-LF	ATCGCAAGCATGCTGCCGTT	Validation of single exchange of *baeC* gene
baeC-dan-RR	CATCTTTTTACCGGTGACAC	Validation of single exchange of *baeC* gene
baeD-dan-LF	AAACCGTGAAAATTCCTCCT	Validation of single exchange of *baeD* gene
baeD-dan-RR	AGTATGGCCACTGACGGAAA	Validation of single exchange of *baeD* gene
baeE-dan-LF	TCGGTCGATAATCGGCTTTG	Validation of single exchange of *baeE* gene
baeE-dan-RR	GAGCGCTGAGGATGCTTTAG	Validation of single exchange of *baeE* gene
pksG-dan-LF	AGCGGATGACTGCTTTTTTC	Validation of single exchange of *pksG* gene
pksG-dan-RR	TCAAAAGCCGAACGGAATCC	Validation of single exchange of *pksG* gene
C-*sfp*-F	CGGGATCCCTTTCGTCTGATATGA	Amplification of *sfp* gene
C-*sfp*-R	GCTCTAGAACAAAAAAGGAGGGGG	Amplification of *sfp* gene
C-*acpk*-F	CGGGATCCCATGACATCAAGGTAT	Amplification of *acpk* gene
C-*acpk*-R	GCTCTAGAGAGACGGCCTCGCTTT	Amplification of *acpk* gene
C-*pksG_2*-F	CGGGATCCGACGTTTATCGTTTCA	Amplification of *pksG* gene
C-*pksG_2*-R	GCTCTAGACATATTGGAGGCGAAG	Amplification of *pksG*_gene
27-F	AGAGTTTGATCCTGGCTCAG	Amplification of 16S rDNA
1492-R	GGTTACCTTGTTACGACTT	Amplification of 16S rDNA

Note: "GGATCC" represents the *BamH* I cleavage site sequence, "TCTAGA" represents the *Xba* I cleavage site sequence.

Table S4 Primers for qRT-PCR used in this study

Name of primer	Primer sequence（5'-3'）	Purpose
PR1-qF	GGAGCTACGCAGAACAACTAAGA	Amplification of *PR1* gene
PR1-qR	CCCACGAGGATCATAGTTGCAACTGA	Amplification of *PR1* gene
coi1-qF	GATTCCATCGTCCCACTTTC	Amplification of *coi1* gene
coi1-qR	CATAACCACCAGAGTGAGAAG	Amplification of *coi1* gene
etr1-qF	GGTCGCTGTGAGGGTTCCGCTTCT	Amplification of *etr1* gene
etr1-qR	CATGCCATTGCCTTGCACTATCTG	Amplification of *etr1* gene
fliC-qF	GAACGCCAACGGTGCGAACT	Amplification of *fliC* gene
fliC-qR	GCTTCGACGACCTTCCAATAC	Amplification of *fliC* gene
BETA-TUB-qF	GAGGGAGCCATTGACAACATCTT	Amplification of *beta-Tub* gene
BETA-TUB-qR	GCGAACAGTTCACAGCTATGTTCA	Amplification of *beta-Tub* gene

Figure S1 Relative abundances of different nodes in endophytic networks of healthy and diseased plants. **(A)** Proportion of nodes belonging to different phylogenetic groups; **(B)** Venn diagrams indicating the number of nodes shared and not shared by two networks. ED: diseased plant network. EH: healthy plant network.

(A)

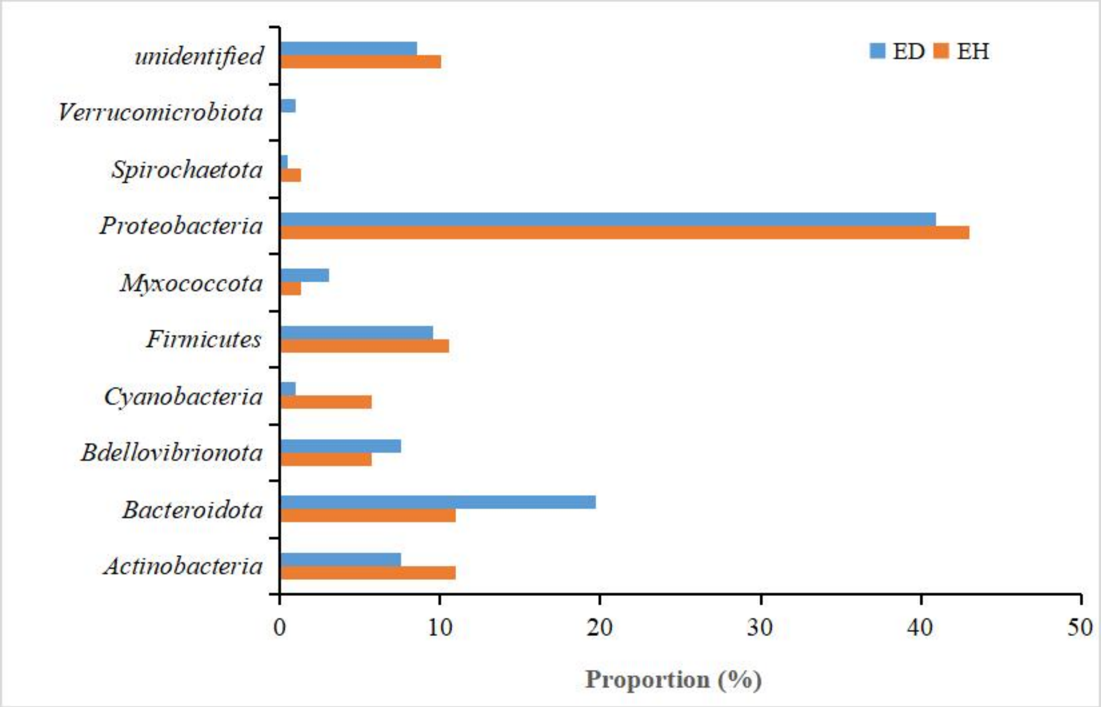

(B)

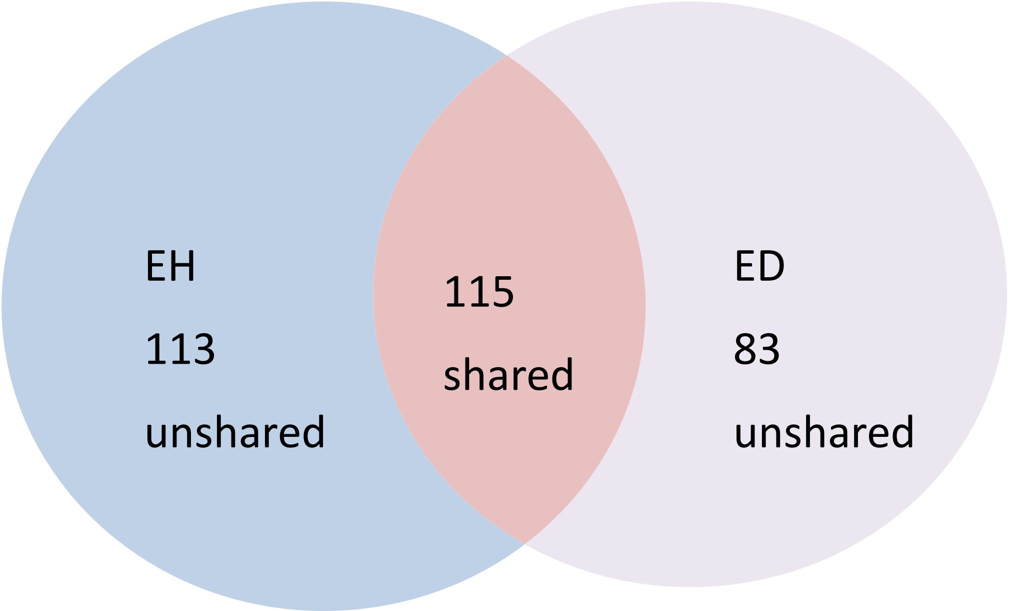

Figure S2 Screening nitrogen-fixing bacteria. (A) Detecting growth of bacteria in Ashby medium. **(B)** Electrophoresis detection of the first PCR product of gene *nifH*. **(C)** Electrophoresis detection of the second PCR product of gene *nifH*. M. Molecular weight marker, with molecular weight were 2000 bp, 1000 bp, 750 bp, 500 bp, 250 bp and 100 bp from top to bottom, respectively.

(A)

P. rodasii E1 *K. intermedia* E2 *P. putida* E3 *P. putida* E4 *K. intermedia* E5 *B. petrii* E6 *P. poae* E7

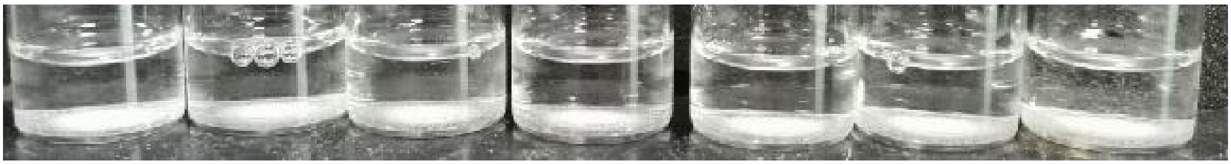

B. subtilis E8 *B. velezensis* E9 *B. velezensis* E10 *B. cereus* E11 *B. altitudinis* E12 *P. extremorientalis* E13 *B. aryabhattai* E14

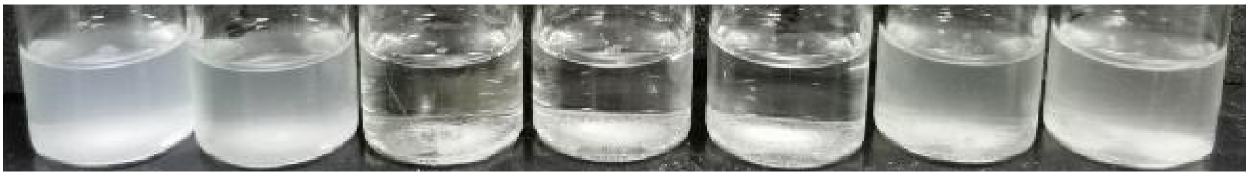

P. simiae E15 *P. brassicacearum* E16 *P. poae* E17 *P. simiae* E18 *S. vinaceus* E19 *P. putida* E20 *M. maritypicum* E21

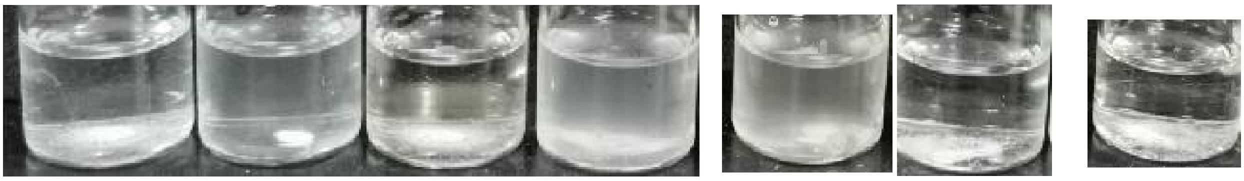

P. antarctica E22 P. putida E23 P. fluorescens E24 P. trivialis E25 P. extremorientalis E26 P. extremorientalis E27 S. thermophilus E28

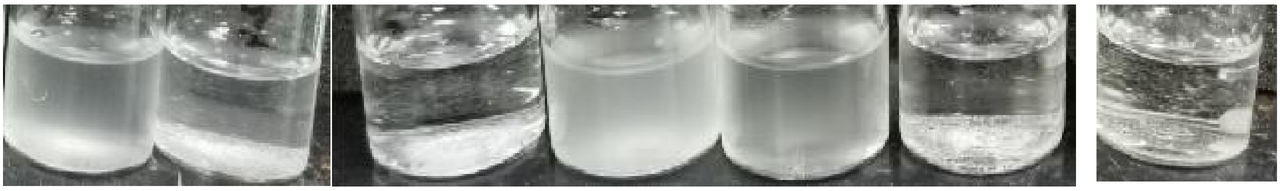

Pseudomonas putida E44 *Pseudomonas geniculata* E53 *A. marplatensis* E54

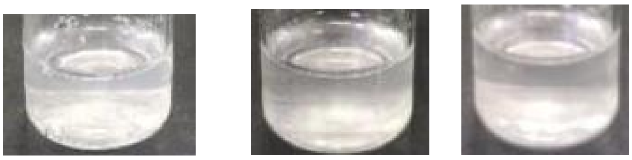

((B)

E8 E9 E13 E14 E18 E22 E25 E26 E19 M

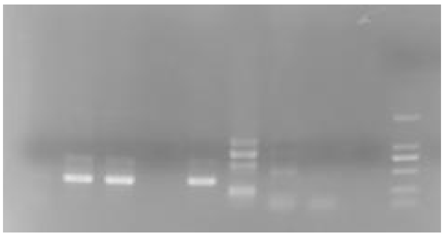

(C)

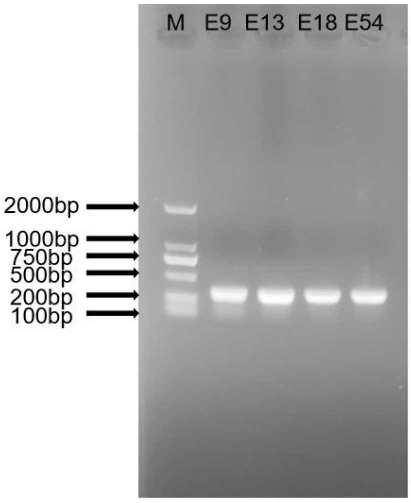

Figure S3 Detecting the biofilm formed by endophytic bacteria. All data shown are average values with standard deviations from triplicate experiments.

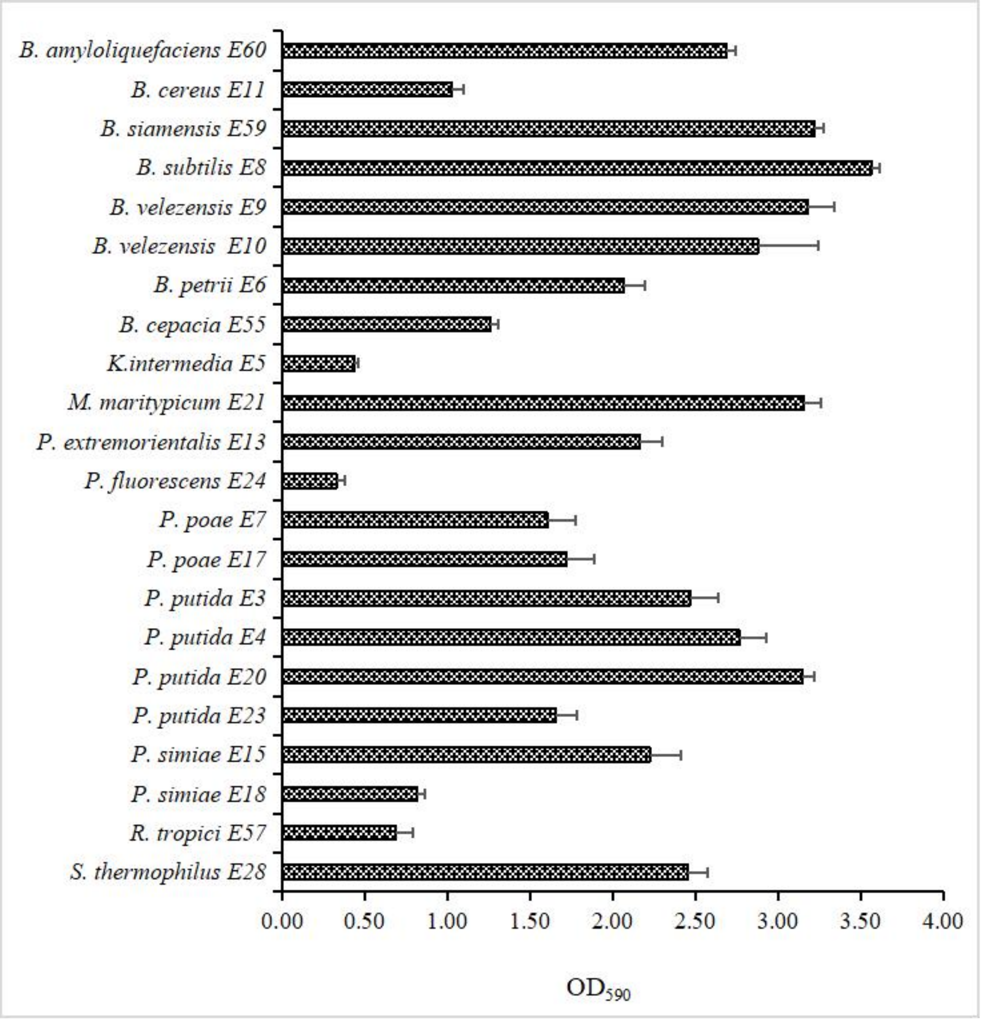

Figure S4 Construction of the knockout strain. A: Construction of mutant △*sfp*, left is the construction of the recombinant plasmid, lane 1: The correct transformant, about 1330 bp in size. Right is the double exchange validation results, lane 3: The strain E9 with a deletion of the *sfp* gene, about 1330 bp in size. B: Construction of mutant Δ*srfA*, left is the construction of the recombinant plasmid, lane 1: The correct transformant, about 1130 bp in size. Right is the double exchange validation results, lane 3: The strain E9 with a deletion of the *srfA* gene, about 1130 bp in size. C: Construction of mutant △*ymfD*, left is the construction of the recombinant plasmid, lane 5: The correct transformant, about 1190 bp in size. Right is the double exchange validation results, lane 1: The strain E9 with a deletion of the *ymfD* gene, about 1190 bp in size. D: Construction of mutant △*acpK*, left is the construction of the recombinant plasmid, lane 2: The correct transformant, about 1100 bp in size. Right is the double exchange validation results, lane 3: The strain E9 with a deletion of the *acpK* gene, about 1100 bp in size. E: Construction of mutant △*bacE*, left is the construction of the recombinant plasmid, lane 1: The correct transformant, about 1130 bp in size. Right is the double exchange validation results, lane 3: The strain E9 with a deletion of the *bacE* gene, about 1130 bp in size. F: Construction of mutant △*baeD*, left is the construction of the recombinant plasmid, lane 6: The correct transformant, about 1200 bp in size. Right is the double exchange validation results, lane 7: The strain E9 with a deletion of the *baeD* gene, about 1200 bp in size. G: Construction of mutant △*pksG*, left is the construction of the recombinant plasmid, lane 2: The correct transformant, about 1200 bp in size. Right is the double exchange validation results, lane 7: The strain E9 with a deletion of the *pksG* gene, about 1200 bp in size. H: Colony of mutants.

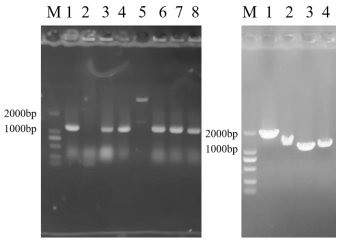

A

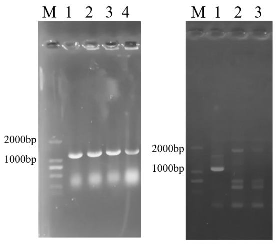

B

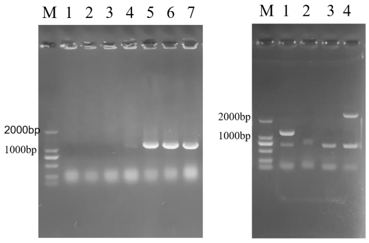

C

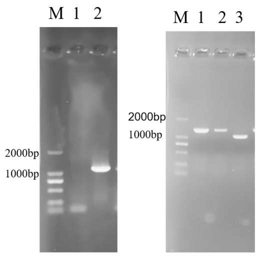

D

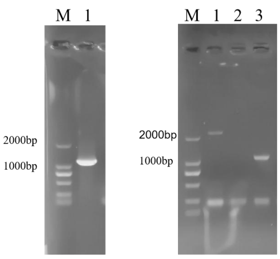

E

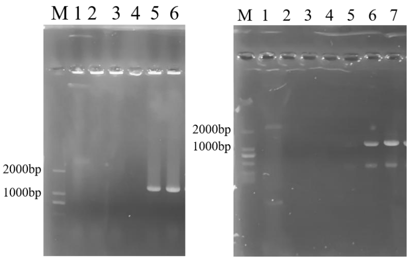

F

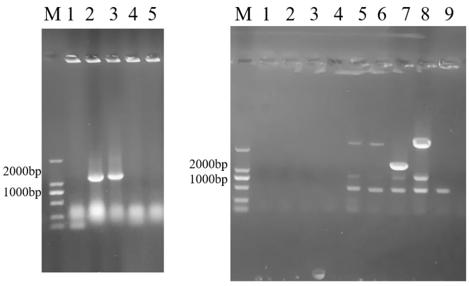

G

H

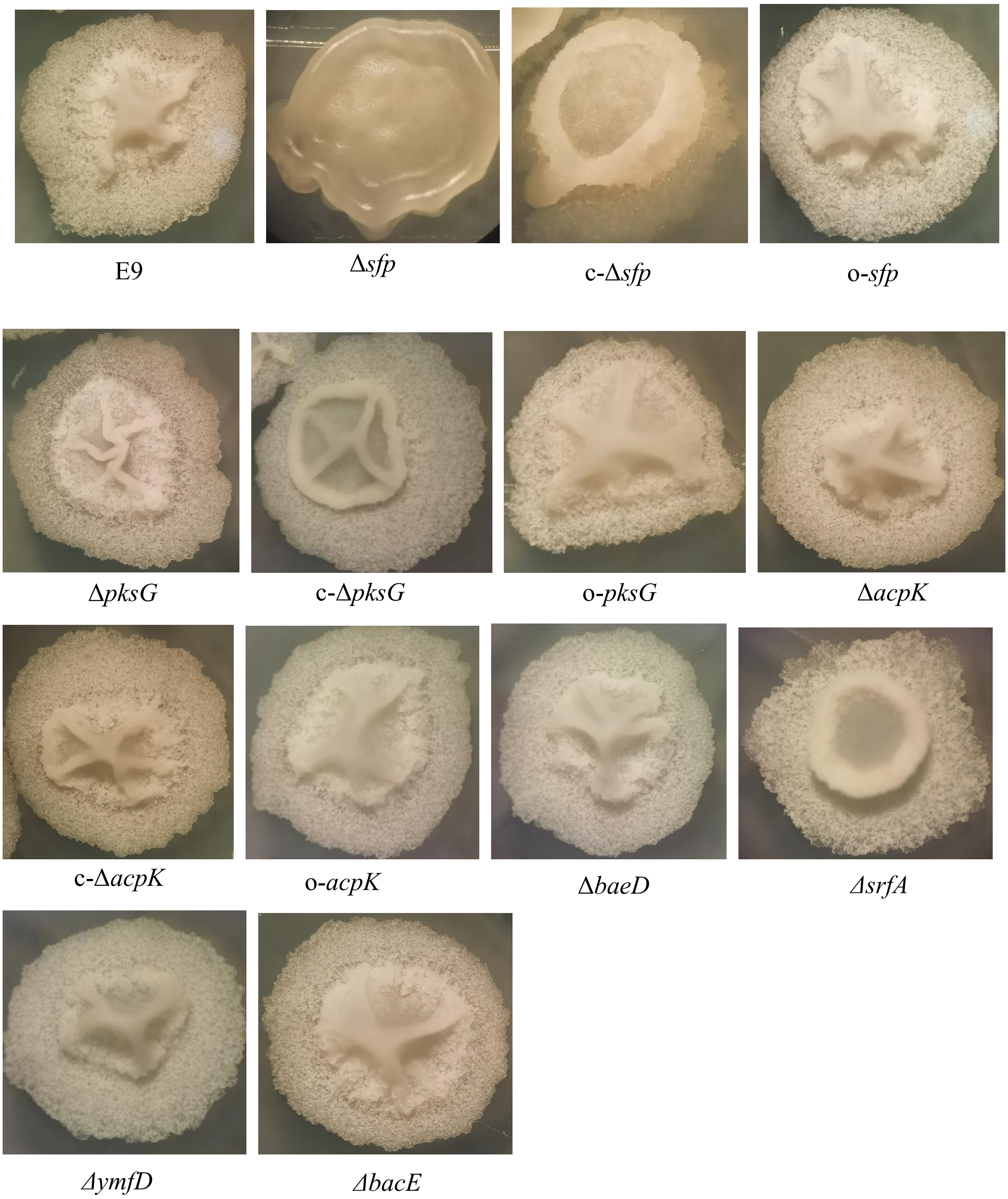
E9
Δ*sfp*
c-Δ*sfp*
o-*sfp*
Δ*pksG*
c-Δ*pksG*
o-*pksG*
Δ*acpK*
c-Δ*acpK*
o-*acpK*
Δ*baeD*
Δ*srfA*
Δ*ymfD*
Δ*bacE*

Figure S5 Endophytic bacteria promoted plant growth and induced systemic resistance. **(A)** Cell number of endophytic bacteria alive in *Arabidopsis* plants. **, * represent significant differences between cell number detected at 28 d and 14 d with that at 0 d at $p < 0.01$ and $p < 0.05$. **(B)** Growth of *A. thaliana* inoculated with endophytic bacteria. Consortium: *A. thaliana* was inoculated with consortium consisted of *P. poae* E7, *B. velezensis* E9, and *P. polymyxa* E29**.** **(C)**, **(D)** Fresh weight and dry weight of plants. Different letters up bar represent significant differences between treatments at $P < 0.05$ level. **(E)**, **(F)**, **(G)** Expression of defense-related genes *PR1*, *coi1*, and *etr1* in *Arabidopsis*. * represent higher than 2.0-fold of control. **(H)** Detecting reactive oxygen species burst.

(A)

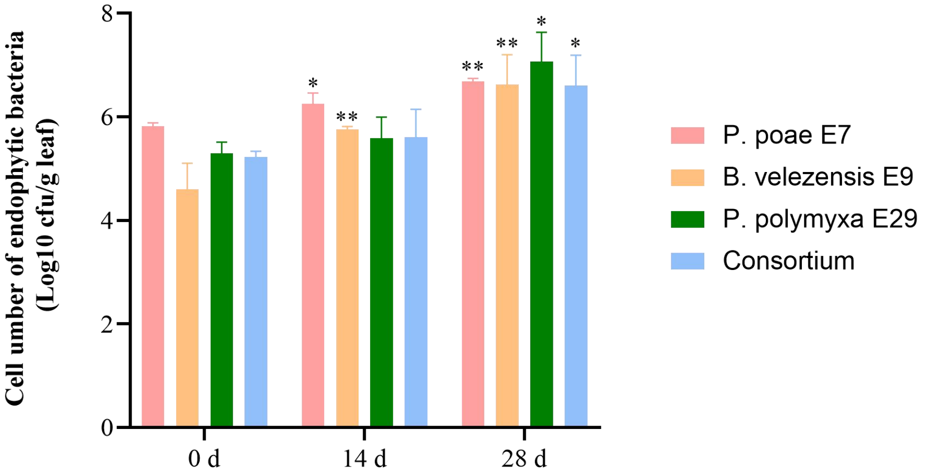

(B)

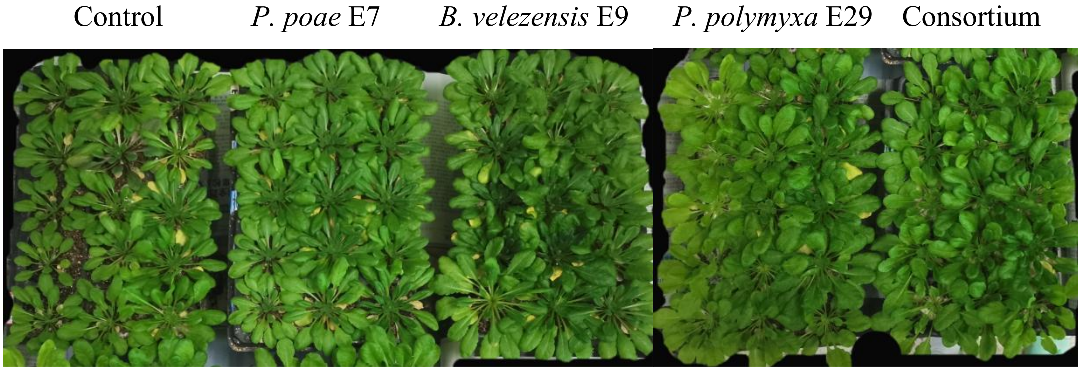

(C)

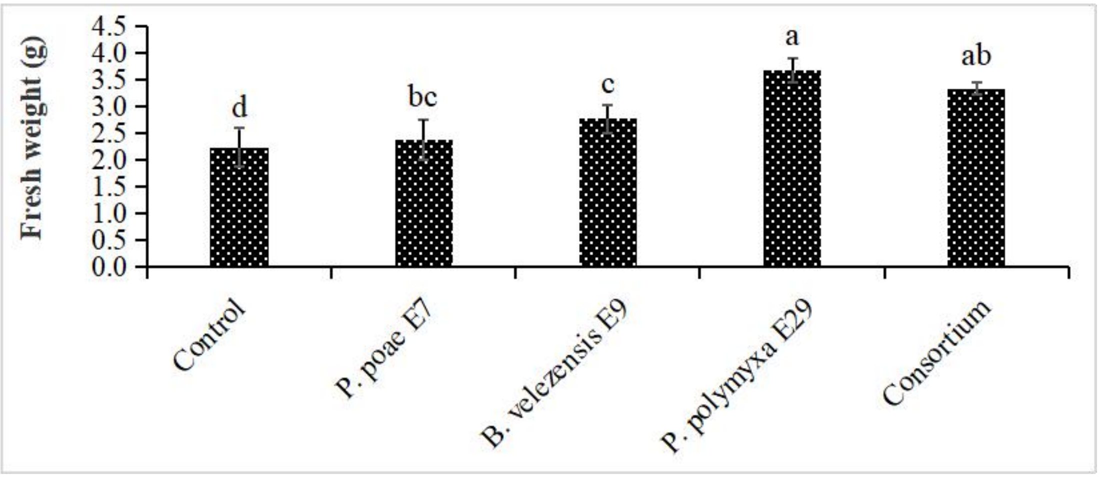

(D)

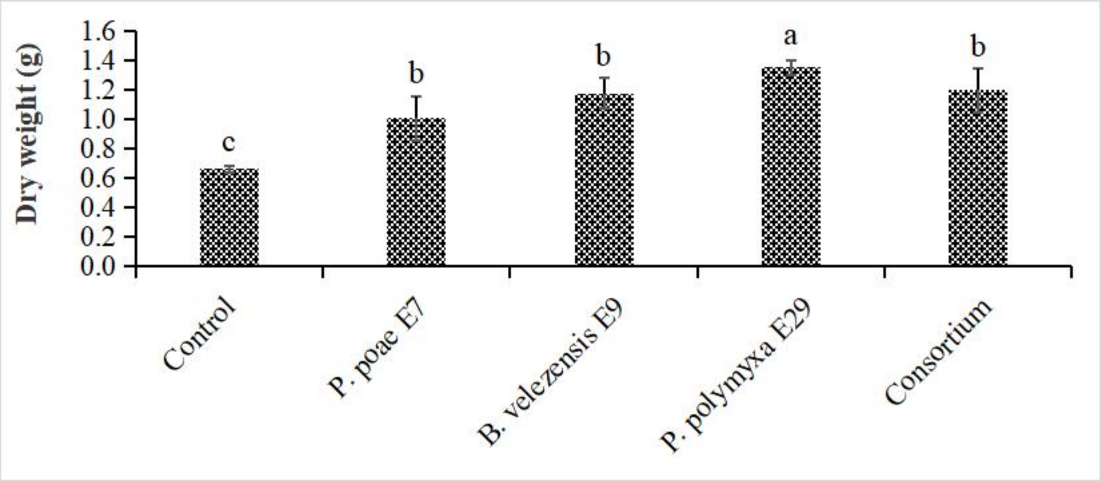

Dry weight (g)
1.6
1.4
1.2
1.0
0.8
0.6
0.4
0.2
0.0
c
b
b
a
b
Control
P. poae E7
B. velezensis E9
P. polymyxa E29
Consortium

(E)

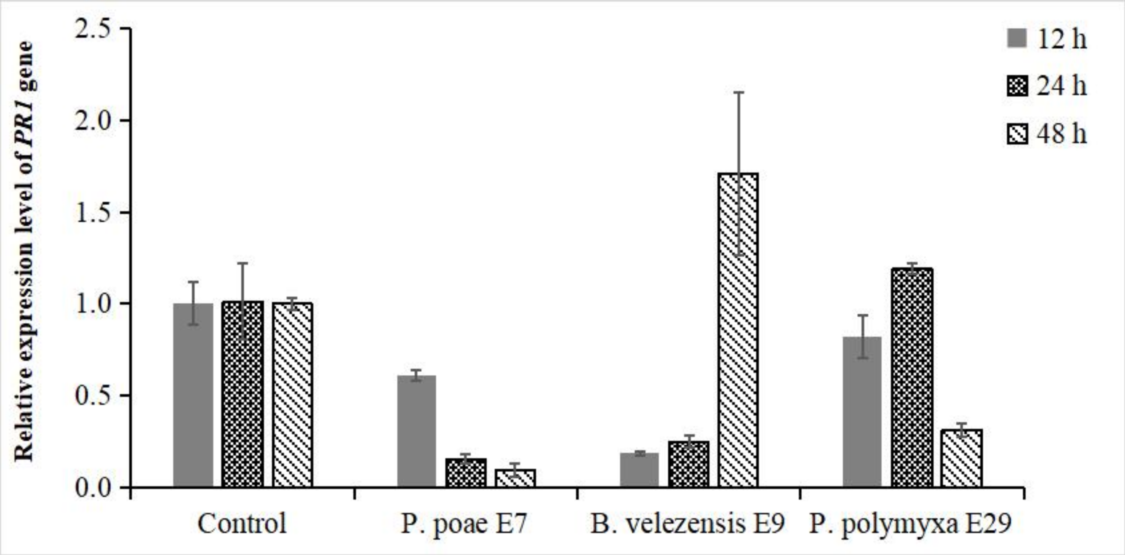

Relative expression level of *PR1* gene
2.5
2.0
1.5
1.0
0.5
0.0
12 h
24 h
48 h
Control
P. poae E7
B. velezensis E9
P. polymyxa E29

(F)

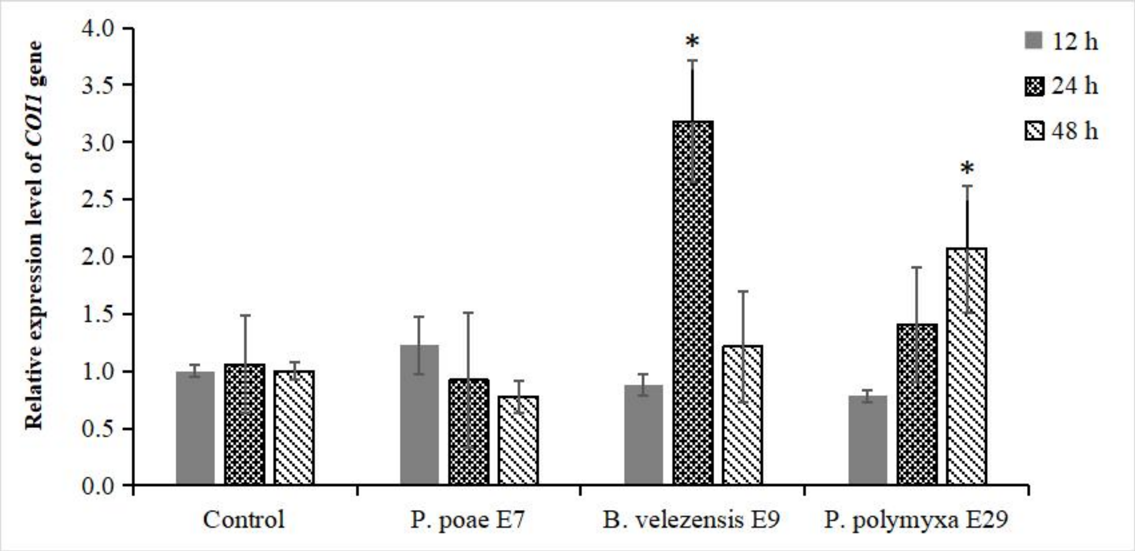

Relative expression level of *COI1* gene
4.0
3.5
3.0
2.5
2.0
1.5
1.0
0.5
0.0
*
*
12 h
24 h
48 h
Control
P. poae E7
B. velezensis E9
P. polymyxa E29

(G)

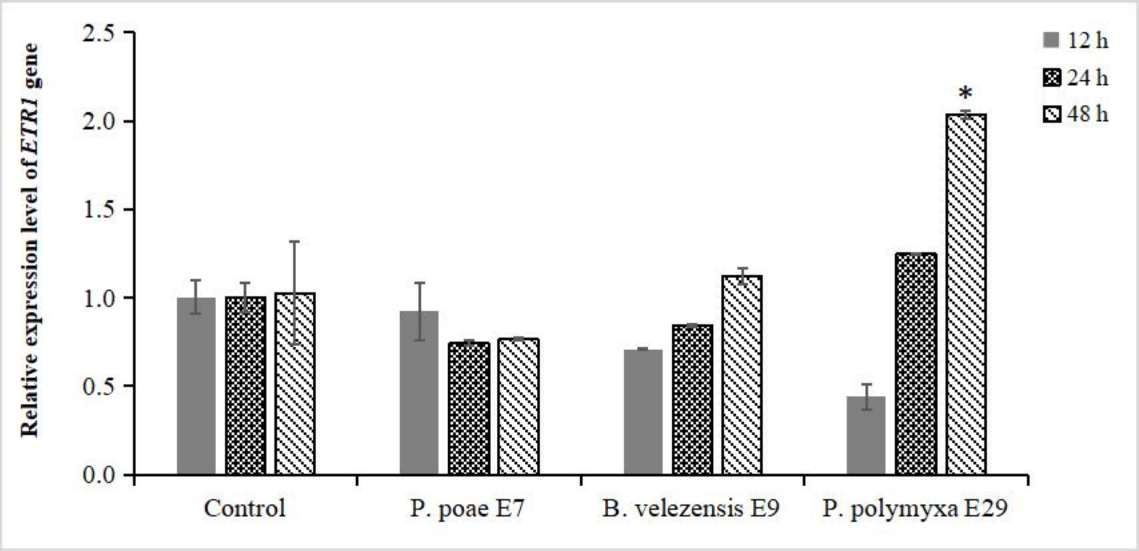

Relative expression level of *ETR1* gene
2.5
2.0
1.5
1.0
0.5
0.0
12 h
24 h
48 h
*
Control
P. poae E7
B. velezensis E9
P. polymyxa E29

(H)

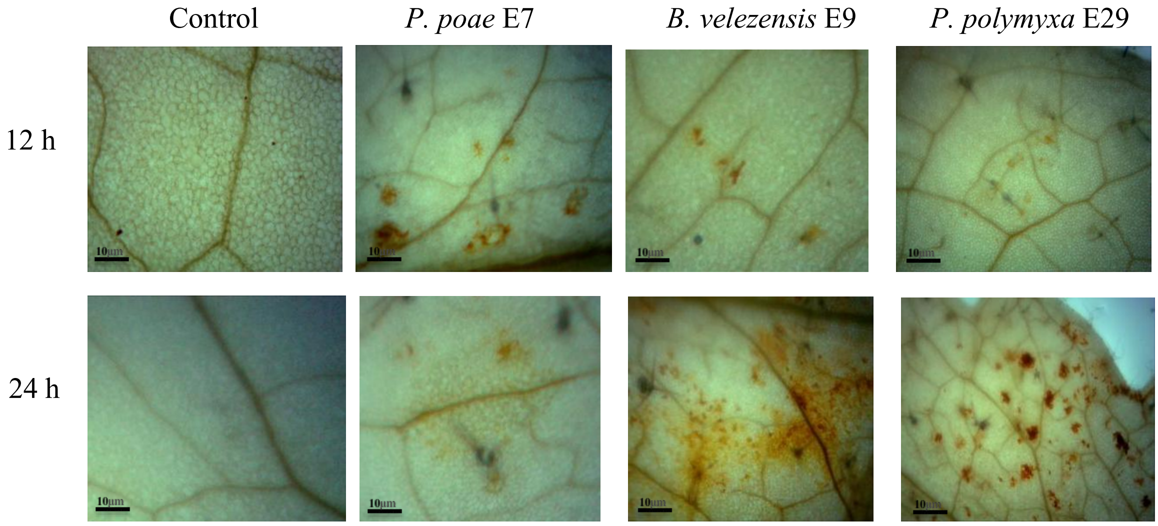

Control
P. poae E7
B. velezensis E9
P. polymyxa E29
12 h
24 h

Figure S6 Comparison of disease severity index in healthy (EH) and bacterial wilt diseased (ED) tobacco plants. (A) Disease severity index. **(B)** Healthy tobacco plant with disease severity index of 0 grows healthy in the EH field. **(C)** Tobacco plant with disease severity index of 9 is completely withered in the ED field. *** represent significant ($P < 0.001$) difference between two groups.

(A) **(B)** **(C)**

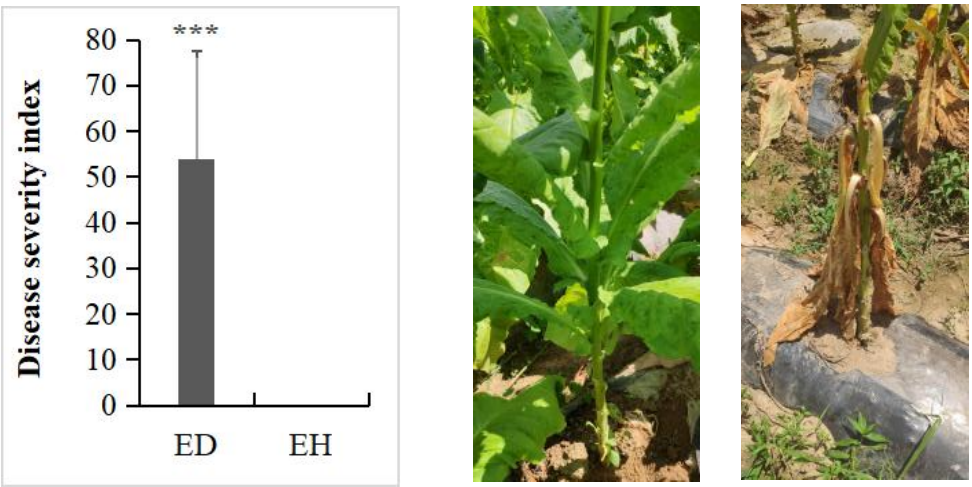

Supplement: Supplemental file 1 — Supplemental material. Download spectrum.02031-22-s0001.pdf, PDF file, 1.9 MB [file spectrum.02031-22-s0001.pdf]
